# *Bifidobacterium asteroides* PRL2011 Genome Analysis Reveals Clues for Colonization of the Insect Gut

**DOI:** 10.1371/journal.pone.0044229

**Published:** 2012-09-20

**Authors:** Francesca Bottacini, Christian Milani, Francesca Turroni, Borja Sánchez, Elena Foroni, Sabrina Duranti, Fausta Serafini, Alice Viappiani, Francesco Strati, Alberto Ferrarini, Massimo Delledonne, Bernard Henrissat, Pedro Coutinho, Gerald F. Fitzgerald, Abelardo Margolles, Douwe van Sinderen, Marco Ventura

**Affiliations:** 1 Alimentary Pharmabiotic Centre and Department of Microbiology, Bioscience Institute, National University of Ireland, Cork, Ireland; 2 Laboratory of Probiogenomics, Department of Genetics, Biology of Microorganisms, Anthropology and Evolution University of Parma, Parma, Italy; 3 Departamento de Microbiologia y Bioquimica de Productos Lacteos, IPLA – CSIC, Villaviciosa, Asturias, Spain; 4 Functional Genomic Center, Department of Biotechnology, University of Verona, Verona, Italy; 5 Glycogenomics, Databases and Bioinformatics, Architecture et Fonction des Macromolécules Biologiques, Universités Aix-Marseille, Marseille, France; University of Vienna, Austria

## Abstract

Bifidobacteria are known as anaerobic/microaerophilic and fermentative microorganisms, which commonly inhabit the gastrointestinal tract of various animals and insects. Analysis of the 2,167,301 bp genome of *Bifidobacterium asteroides* PRL2011, a strain isolated from the hindgut of *Apis mellifera* var. *ligustica*, commonly known as the honey bee, revealed its predicted capability for respiratory metabolism. Conservation of the latter gene clusters in various *B. asteroides* strains enforces the notion that respiration is a common metabolic feature of this ancient bifidobacterial species, which has been lost in currently known mammal-derived *Bifidobacterium* species. In fact, phylogenomic based analyses suggested an ancient origin of *B. asteroides* and indicates it as an ancestor of the genus *Bifidobacterium*. Furthermore, the *B. asteroides* PRL2011 genome encodes various enzymes for coping with toxic products that arise as a result of oxygen-mediated respiration.

## Introduction

Bifidobacteria are common commensals of the mammalian gut, where they are considered to contribute to the host's metabolism and physiology [1]–[8]. However, bifidobacteria have also been isolated from the intestine of invertebrates such as insects [9], [10]. In microbiomic surveys of the hindgut of social insects (honey bees, wasps and bumble bees) *Bifidobacterium asteroides* was the most frequently isolated *Bifidobacterium* species [11], [12], [13]. Such and other findings support the idea that members of this genus enjoy a wide-spread distribution in a large variety of hosts, including animals that raise their offspring by parental care (e.g., mammals, birds, social insects), and it may thus be that such an ecological distribution is the consequence of direct transmission of bifidobacterial cells from parent/carer to offspring [14]. The gut of mammals/birds is believed to be an anaerobic body compartment and consequently members of the genus *Bifidobacterium* and many other gut commensals exhibit a strict anaerobic/microaerophilic metabolism [15]. However, the previously presumed anaerobic gut commensal *Bacteroides fragilis* can perform respiration at low oxygen levels [16]. Identification of an oxygen-dependent respiratory chain that allows this microorganism to use an alternative metabolic pathway has prompted the identification of a group of bacterial anaerobes, that can benefit from nanomolar concentrations of oxygen [16]. In contrast to obligate anaerobes, facultative anaerobes, such as *Escherichia coli*, grow most rapidly when respiring in the presence of oxygen, and in the absence of oxygen will switch to either anaerobic respiration, or to fermentation if no alternative electron acceptors are available [17].

The toxic effects of oxygen on bifidobacterial growth and survival have been covered by several studies [18], [19]. Bifidobacterial growth is reportedly inhibited by oxygen, and prolonged aeration of bifidobacterial cultures can lead to cell death and DNA degradation [20]. Within the genus *Bifidobacterium* only a few species, i.e. *Bifidobacterium animalis* subsp. *lactis* and *Bifidobacterium psychraerophilum* have been found to tolerate oxygen [21], [22], but at a lower level compared to that observed for the insect isolate *B. asteroides* [10]. In fact, growth of *B. animalis* subsp. *lactis* and *B. psychraerophilum* species is arrested at atmospheric levels(∼20%) of oxygen, whereas this level would still allow growth in the case of *B. asteroides* [10], [18], [23].

Genome sequencing of bifidobacteria have so far been motivated predominantly by the interest to unravel the intricate interactions between the bacterium and its mammalian host [1], [24]–[30]. Here, we report on the genome analysis of *B. asteroides* PRL2011, revealing predicted metabolic traits that underpin the first reported case of respiration within the genus *Bifidobacterium*.

## Results and Discussion

### General genome features

The chromosome of *B. asteroides* PRL2011 (accession number CP003325) was determined to consist of 2,167,301 base pairs, with a G+C content of 60.49%, which is similar to that of other bifidobacterial genomes [24]–[29], and falling within the typical range of *Actinobacteria* [15]. Furthermore, PRL2011 contains an extra chromosomal element consisting of 2,111 bp. Salient genome features are presented in Table S1. Clusters of orthologous groups (COGs) analysis of the PRL2011 predicted proteome allowed a functional assignment for 79.7% of the total number of predicted ORFs. Homologs from other bacterial species with unknown function were identified for an additional 20.3% of the *B. asteroides* PRL2011 ORFs, while the remaining 12.8% appear to be unique to *B. asteroides*, a percentage that is considerably higher compared to that obtained for other bifidobacterial genomes [24]–[29]

**Figure 1:**
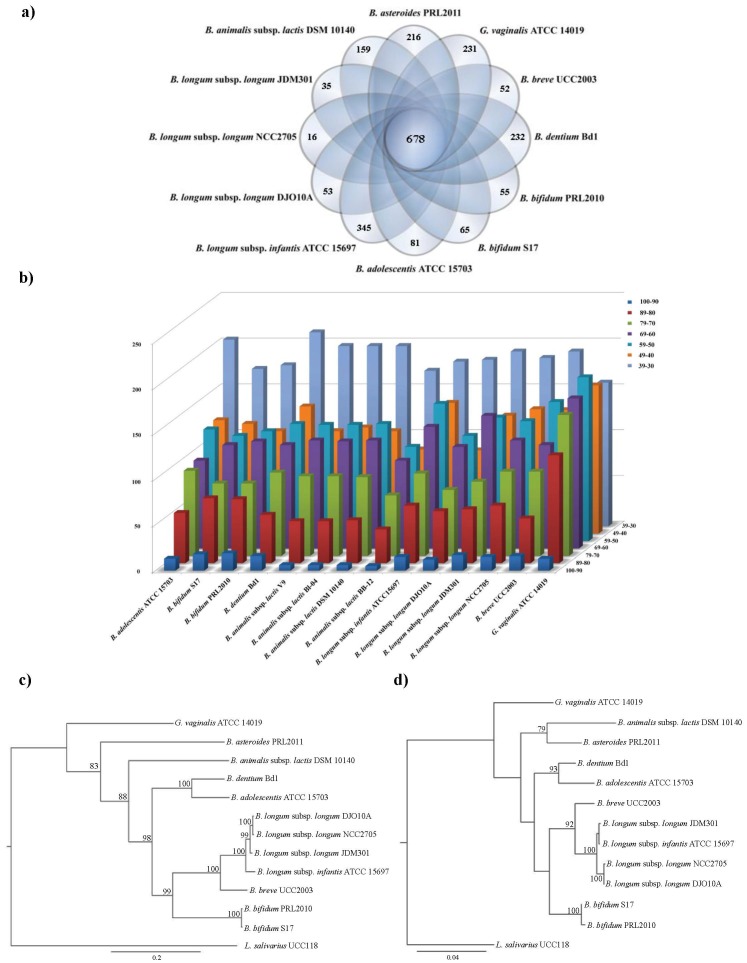
Comparative genomic analysis of *B. asteroides* PRL2011 with other fully sequenced bifidobacterial genomes, as well as *Gardnella vaginalis*. Panel a displays a Venn diagram of homologs shared between sequenced bifidobacterial-*Gardnella* genomes. Panel b shows the percentage of amino acid identity of the top-scoring self-matches for protein-coding genes in the analysed bacteria using the predicted proteome of *B. asteroides* PRL2011 as a reference. For each bacterium, the deduced protein-coding regions for each gene were compared with those derived from the *B. asteroides* PRL2011 genome. Panel c depicts a phylogenetic supertree based on the sequences of *Bifidobacterium-Gardnella* core proteins. Panel d indicates the generated phylogenetic tree based on 16S rRNA gene sequences from the same set of bacteria. Three other members of the *Actinobacteria* phylum, *N. farcinia*, *T. whipplei* and *L. xyli*, were also included in the analyses depicted in panels c and d, while the trees were rooted using *L. salivarius* as outgroup.

A comparative study was undertaken to find orthologs between the *B. asteroides* PRL2011 ORFs and all other currently available and fully sequenced *Bifidobacteriaceae* genomes. The results obtained revealed 678 putative orthologs that were shared among these genomes (Fig. 1A). The most common functional class represented by these assumed core proteins was, as expected, the class corresponding to housekeeping functions.

Notably, about 40% of the *B. asteroides* unique proteins were functionally categorized (Table S2) according to COG assignment in the carbohydrate metabolism and transport family (15.7%), inorganic ion transport and metabolism (11.6%) and in energy production and conversion family (5.8%). This suggests that *B. asteroides* possesses a number of metabolic features that are unique among the so far characterized bifidobacteria (see below).

Among these *B. asteroides* PRL2011-specific proteins, we in particular noted the presence of 19 nearly identical copies of a gene predicted to specify an extracellular protein with RCC1-domains and an anonymous *Listeria-Bacteroides* domain (PFAM09479), which displays a β-propeller architecture consisting of modular β-sheet building blocks that are circularly arranged [31], [32] (Fig. S7). Such proteins, to which no distinct function has yet been assigned, are widely distributed in eukaryotes, yet have only occasionally been detected in bacteria [33]. The RCC1 proteins encoded by the PRL2011 genome were shown to be phylogenetically closer to homologs of bacterial origin rather than those found in eukaroytes (Fig. S7).

To investigate the phylogenetic position of *B. asteroides* PRL2011 within the genus *Bifidobacterium* [15], while also comparing this to *Gardnerella vaginalis*, which is a closely related member of the family *Bifidobacteriaceae*, a phylogenomic analysis was performed that was based on 471 protein sequences representing the minimal core protein set of the *Bifidobacterium*/*Gardnerella* genera, and exclusively encompassing housekeeping genes that are present in a single copy [34]. Interestingly, the resulting neighbour-joining tree, which has been constructed according to a previous described method [35], revealed a clear evolutionary split of *B. asteroides* from a distinct phylogenetic cluster that consists of the other currently available bifidobacterial genomes (Fig. 1C–D). This apparent ancient evolutionary diversification of the *B. asteroides* PRL2011 genome is corroborated by the low abundance of encoded gene products with high similarity to proteins that are encoded by currently available bifidobacterial genomes: of the predicted 1686 proteins putatively specified by *B. asteroides* PRL2011, only 12 share >90% amino acid identity with proteins encoded by other bifidobacterial genomes, while 435 share an identity level that ranges between 50% and 30% (Fig. 1B).

### Metabolism and transport of *B. asteroids*

Homologs of all enzymes necessary for the fermentation of glucose and fructose to lactic acid and acetate through the characteristic “fructose-6-phosphate shunt” [36], as well as enzymes for a partial Embden-Meyerhoff pathway were annotated in the *B. asteroides* PRL2011 chromosome.

Interestingly, the chromosome of PRL2011 contains a genetic locus encoding a malate carrier, a malolactic enzyme and a possible regulator *mle*R (BAST_0548–BAST_0550) (Fig. S6), representing key players of the so-called malolactate fermentation pathway, which is commonly employed by lactic acid bacteria [37]. Notably, this DNA region is absent in all other so far sequenced bifidobacterial genomes with the exception of the *B. dentium* Bd1 chromosome. The malolactate fermentation pathway is responsible for the conversion of malic acid to lactate, which is then removed from the cells by a malate antiporter, thus generating a proton gradient [38]. PRL2011 is predicted to encode two such malate antiporters, one in the putative malate locus (BAST_0550), while another is positioned elsewhere (BAST_0038).

Like other bifidobacteria, the genome of PRL2011 encode almost 10% of genes dedicated to the carbohydrate metabolism, thus reinforcing the notion that this COG category is highly represented in all bifidobacterial genomes, including those from insect gut origin.

Genomic data combined with carbohydrate-fermentation experiments suggests that *B. asteroides* is capable of metabolizing a broader range of simple carbohydrates than any other tested bifidobacterial species (Fig. S1), many of which (e.g., glucose and fructose) are presumed to be abundant in the honeybee hind gut, representing the natural ecological niche of the *B. asteroides* species [39]. Classification in accordance to the Carbohydrate Active Enzymes (CAZy) system of Coutinho & Henrissat (1999) revealed that the *B. asteroides* PRL2011 genome specifies 72 carbohydrate-active proteins including glycoside hydrolases (GH), glycosyltransferases (GT) and glycosyl esterases (CE), which are distributed in 22 GH families, seven GT and two CE families (Fig. S1).

The genome of *B. asteroides* PRL2011 contains 44 genes that are predicted to encode components of ABC-type transporters, and, as based on the TC database [40], 14 of these proteins are predicted to be involved in the internalization of carbohydrates, eight in the uptake of amino acids/peptides, four in the internalization of metals, nine in conferring various resistances, while nine have no defined function (Table S3). Furthermore, the transporter arsenal of PRL2011 includes one putative phosphoenolpyruvate-phosphotransferase system (PEP-PTS), four proteins that are predicted to be responsible for metal ion uptake and a further eight that represent suspected transporters for amino acids.

Similar to other bifidobacteria, complete biosynthetic pathways for purines and pyrimidines from glutamine, as well as for folate (vitamin B9) were annotated in the genome of *B. asteroides* PRL2011, while it did not encode known biosynthetic pathways for other B vitamins, such as riboflavin and thiamine, which are also variably distributed in published bifidobacterial genomes [25], [27], [28], [29], [41]. We also found evidence of complete amino acid biosynthetic pathways for most amino acids.

The *B. asteroides* PRL2011 genome contains several conventional mobilome candidates that may have been acquired through Horizontal Gene Transfer (HGT) (see for details Text S1, Fig. S5), and that may have provided ecological advantages, while also impacting on chromosome structure and function [15].

### Growth of bifidobacteria in presence of oxygen

In contrast to fermentative carbohydrate metabolism, which has been extensively described in bifidobacteria [42], [43], the utilization of oxygen as a terminal electron acceptor through a respiratory metabolic pathway has not been identified or even suspected in bifidobacteria.

**Figure 2:**
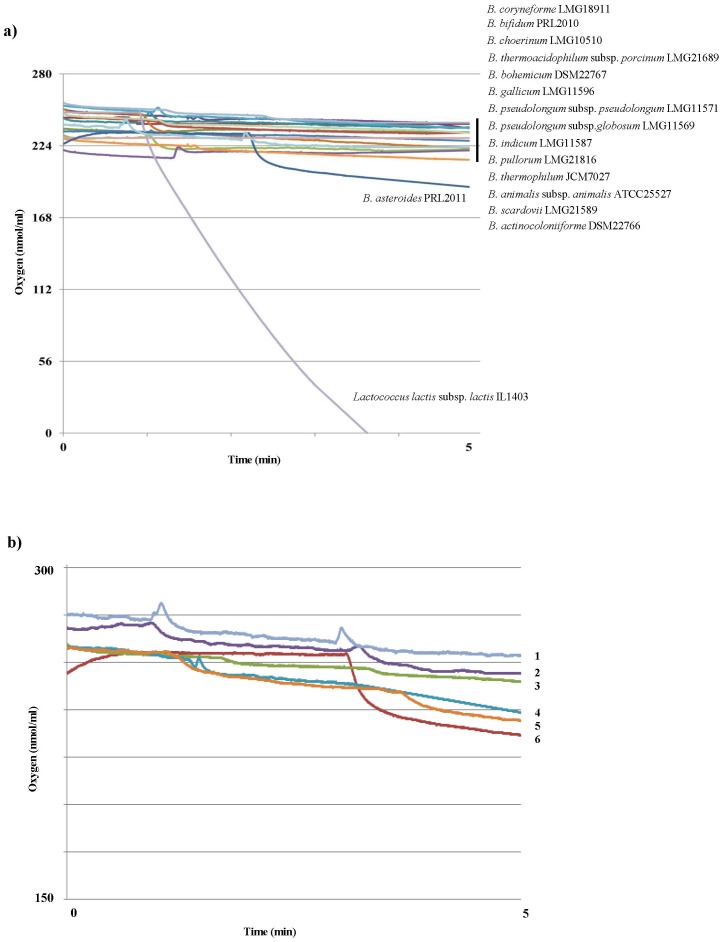
Oxygen consumption of bifidobacteria. Panel a represents the oxygen uptake of different bifidobacterial species. Bifidobacteria were grown to mid-log phase in the absence of oxygen and placed in an oxygraph chamber. *Lactococcus lactis* subsp. *lactis* IL1403 was used as positive control. Panel b, shows the oxygen utilization of different *B. asteroides* cultures grown in the presence of 6.54–6.60 ppm of oxygen to mid-log phase in MRS plus succinate 1% as unique carbon source and without cysteine (curve 1), in MRS plus glucose 2% and cysteine (curve 2), in MRS plus citrate 1% as unique carbon source and without cysteine (curve 3), in MRS plus glucose 2% without cysteine (curve 4) in MRS with glucose 2% without cysteine and hemin 0.5 µg/ml (curve 5), and in MRS with glucose 2% without cysteine and protoporphyrin 10 ug/ml (curve 6).

Oxygen is reported to generally elicit a toxic effect on bifidobacterial growth [18]. Although various bifidobacterial strains have been evaluated for their ability to grow in the presence of oxygen [15], only a small number of strains, all belonging to the *B. asteroides* species, were capable of growing under aerobic conditions [10]. When oxygen consumption by various bifidobacteria was analyzed, only *B. asteroides* PRL2011 was able to use 6.2 nmol/min of O_2_ per mg dry cells, whereas no O_2_ consumption was detected for any of the other bifidobacterial strains tested including other species phylogenetic related to the *B. asteroides* species such as *B. coryneforme*, *B. indicum*, *B. actinocoloniforme* and *B. bohemicum* (Fig. 2). This finding represents a clear hint that respiration does take place in *B. asteroides* PRL2011, but for unknown reasons at a much lower level compared to other bacteria for which respiration as an alternative metabolic pathway to fermentation has been observed, e.g. *Lactococcus lactis*. It is possible that the lower level of oxygenation of the insect gut has resulted in the evolution of a low respiratory level of *B. asteroides*, similar to that observed for nanoaerobes [16]. However, it may also be that we simply did not apply the correct environmental parameters to fully induce respiratory metabolism in *B. asteroides* PRL2011.

### Genomics of putative respiratory metabolism in *B. asteroids*

Respiration requires a functional electron transfer chain, consisting of a number of primary dehydrogenases, terminal reductases/oxidases and different cytochrome types, the choice of which being dependent on performing either aerobic or anaerobic respiration, and the availability of particular electron donors and acceptors [44]. A large proportion of the ‘unique’ genes (i.e. unique compared to other available bifidobacterial genomes) of *B. asteroides* PRL2011 is predicted to encode enzymes for aerobic respiration (Table S2). Unlike *E. coli*, the *B. asteroides* PRL2011 genome does not appear to specify cytochrome types other than oxidase type *d*. This cytochrome type possesses a high oxygen affinity and in *E. coli* is mostly expressed at low oxygen levels [45], [46].

**Figure 3:**
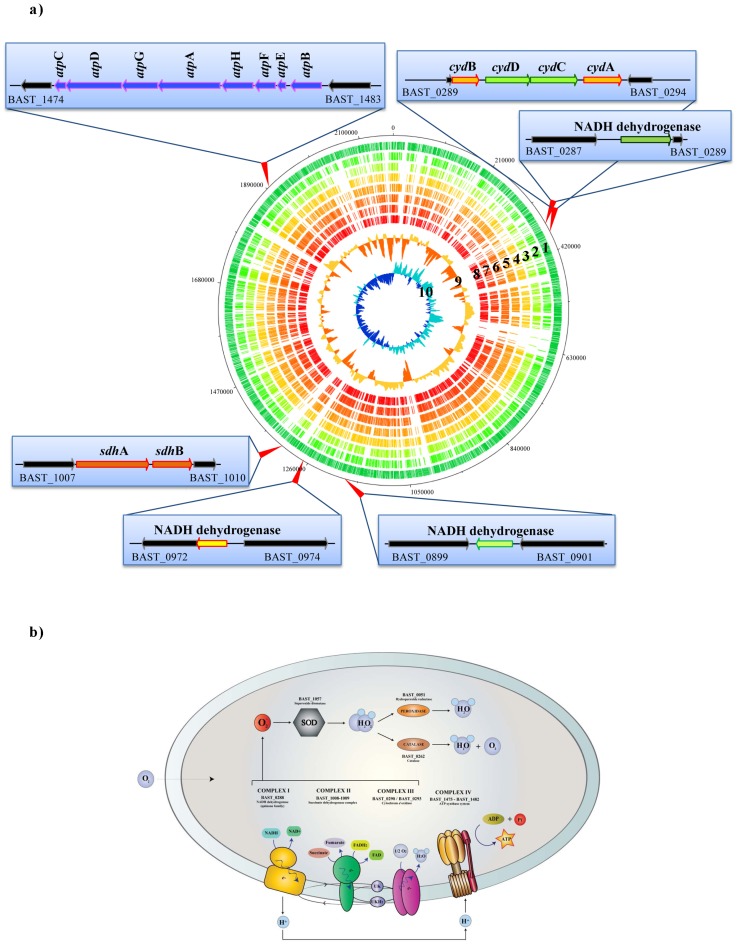
The genes and encoded products of *B. asteroides* PRL2011 predicted to be involved in respiration and oxygen damage. Panel a represents a circular genome atlas of *B. asteroides* PRL2011 (circle 1) with mapped orthologs (defined as reciprocal best FastA hits with more than 30% identity over at least 80% of both protein lengths) in seven publicly available *Bifidobacterium* genomes. From the outer circle, circle (2) shows *B. breve* UCC2003, circle (3) *B. animalis* subsp. *lactis* DSM 10140, circle (4) *B. longum* subsp. *infantis* ATCC 15697, circle (5) *B. dentium* Bd1, circle (6) *B. longum* NCC2705, circle (7) *B. bifidum* PRL2010, circle (8) *B. adolescentis* ATCC 15703. Circle(9) illustrates *B. asteroides* PRL2011 G+C% deviation followed by circle (10) that highlights *B. asteroides* PRL2011 GC skew (G−C/G+C). Moreover, the outer insets indicate the main genetic loci encoding enzymes involved in respiratory metabolism, which are mapped on the circular genome atlas of *B. asteroides* PRL2011. Panel b shows a schematic representation of a cell and metabolic pathways for respiration. The different ORFs of *B. asteroides* PRL2011 encoding the presumed enzymes involved in the respiratory chain are indicated.

The predicted *B. asteroides* electron transport chain is assumed to be assembled in the cytoplasmic membrane so that the electron flow can be coupled to proton extrusion across the membrane to generate a proton motive force (Delta pH gradient and electrical potential) essential for generating ATP from ADP and inorganic phosphate by the enzyme ATP synthase. The electron transport chain is composed of four main complexes named COMPLEX I-II-III-IV (Fig. 3), where COMPLEX I and COMPLEX II are primary electron donor species representing the two main entry points into the respiratory chain. In *B. asteroides* PRL2011 COMPLEX I is predicted to be represented by an NADH dehydrogenase and a flavin mononucleotide (FMN) and iron-sulfur cluster-containing protein, where electrons from NADH are transferred to the FMN domain, and then passed on to the membrane-associated quinone carrier molecule with the simultaneous extrusion of protons. COMPLEX II is represented by a succinate dehydrogenase and is composed by two subunits corresponding to a peripheral flavoprotein portion with the active site for succinate (SdhA) and a membrane iron-sulfur portion with an active site for quinone (SdhB), encoded by *sdh*A and *sdh*B genes, respectively (BAST_1008 and BAST_1009). These COMPLEX II genes are conserved in all sequenced bifidobacterial genomes (Fig. S2), probably because their activities are required for certain housekeeping functions (most likely to fulfil biosynthetic tasks). This complex in *E. coli* can use the conversion of succinate to fumarate and FAD to FADH2 to provide electrons to quinones without proton extrusion [44]. Such enzymes are predicted to be linked to the terminal reductase COMPLEX III (cytochrome *d* oxidase) by electron carrier quinones, which are supplying electrons to the enzyme cytochrome oxidase and then to the terminal electron acceptor (oxygen), upon which this is then reduced to water [45]. The suspected *B. asteroides* PRL2011 COMPLEX III subunits are encoded by four genes, including the *cyd*B (BAST_0290) and *cyd*A (BAST_0293), which specify the structural subunits of the cytochrome, as well as the *cyd*D (BAST_0291) and *cyd*C (BAST_0292) (Fig. S3), which code for predicted ABC-type transporter proteins believed to be required for cytochrome assembly [47].

Finally, COMPLEX IV of *B. asteroides* PRL2011 consists of a typical F_1_F_0_-ATPase, which is composed by two sub-complexes, a membrane-extrinsic F_1_ part and a membrane-intrinsic F_0_ part [48], [49]. The primary role of this enzyme in respiring microorganisms is to couple the electrochemical potential difference for H^+^ across the membrane to synthesize ATP from ADP and phosphate [48]. In *B. asteroides* PRL2011 the F_1_F_0_-ATPase is encoded by the *atp* operon (BAST_1475–1482) consisting of eight genes, displaying an overall homology of around 80% with corresponding genes in other bifidobacterial genomes [50].

Additional enzymes that might be involved in the generation of a proton-motive force include a predicted pyruvate oxidase, a lactate dehydrogenase and two glycerol dehydrogenases [51]. Pyruvate oxidase may catalyze the oxidative decarboxylation of pyruvate to acetate and CO_2_ using quinone as the electron acceptor. Its existence in *B. asteroides* PRL2011 is supported by the identification of a *pox*B homologue (BAST_1463) on its genome. Furthermore, inspection of the genome sequences of PRL2011 revealed two genes predicted to encode L-lactate dehydrogenase (*lld*D, BAST_0523) and D-lactate dehydrogenase (*dld*D, BAST_0909). These enzymes are predicted to use quinones as electron acceptor when lactate is used as carbon and energy source. The *B. asteroides* PRL2011 genome encodes two glycerol dehydrogenases (BAST_0304 and BAST_0696), enzymes catalyzing the oxidation of glycerol to dihydroxyacetone and reduces quinone in the cytoplasmic membrane [52].

Genome analysis of PRL2011 allowed the identification of several genes that might be involved in the synthesis of menaquinone from chorismate, the latter being an intermediate in the biosynthesis of aromatic amino acids. These include *men*F (BAST_1596), encoding a isochorismate synthetase, *men*C (BAST_174) coding for an O-Succinylbenzoate synthetase, *men*E (BAST_0720), coding for O-succinylbenzoyl-CoA synthetase, and *men*A (BAST_1279), specifying a 1,4-dydroxy-2-naphthoate prenyltransferase. Similarly, the predicted proteome of *B. asteroides* PRL2011 revealed enzymes that could perform the final three steps of heme biosynthesis from coproporhyrinogen III [51]. Comparative analysis among bifidobacterial genomes display that relative to other characterized bifidobacterial genomes *cydABCD* genes are uniquely present in the chromosome of *B. asteroides*, and that this also applies to other respiratory chain-related genes (Fig. S4). *B. asteroides* seems therefore to represent the only respiring representative of the genus *Bifidobacterium* so far described. Such a conclusion is also corroborated by the ecological niche that it occupies, where the level of oxygen is relatively high [53]. With regards to possible regulation of the *cydABCD* operon, genome analysis of *B. asteroides* PRL2011 revealed the existence of putative homologues of ArcA/B (BAST_0946/0947) and a putative FNR regulator (BAST_0395), which in *E. coli* are involved in controlling the *cyd*D gene [54]. The ArcA/B homologs of *B. asteroides* PRL2011 represent a two-component regulatory system composed of a membrane-linked sensor (ArcB) and a regulatory DNA-binding domain (ArcA), while FNR is a cytoplasmatic O_2_-responsive regulator with a sensory and a regulatory DNA-binding domain [44]. As aerobic respiration would confer a significant advantage to bacteria in the presence of oxygen, it is also true that the resulting reactive oxygen species (ROS) exert a severe toxic effect to proteins, lipids and nucleic acids [55]. Thus, respiring bacteria would also need protection against such ROS [55]. *B. asteroides* PRL2011 genome analysis revealed genes that are predicted to encode several protective enzymes: a catalase (BAST_0262), a superoxide dismutase (BAST_1057), and an *osm*C-like encoding protein potentially induced under oxidative stress (BAST_1188). Furthermore, analysis of the PRL2011 genome did not reveal the existence of any ROS-sensitive iron-sulfur clusters. Notably, compared to other bifidobacteria cultures, PRL2011 cells display a higher survival rate upon exposure to hydrogen peroxide (Text S1). Altogether these findings corroborate our assumption that *B. asteroides* PRL2011contains a particular gene set which allows this bacterium to utilize oxygen by means of a respiratory pathway, and which also provides protection against the toxic effects of aerobic metabolism.

**Figure 4:**
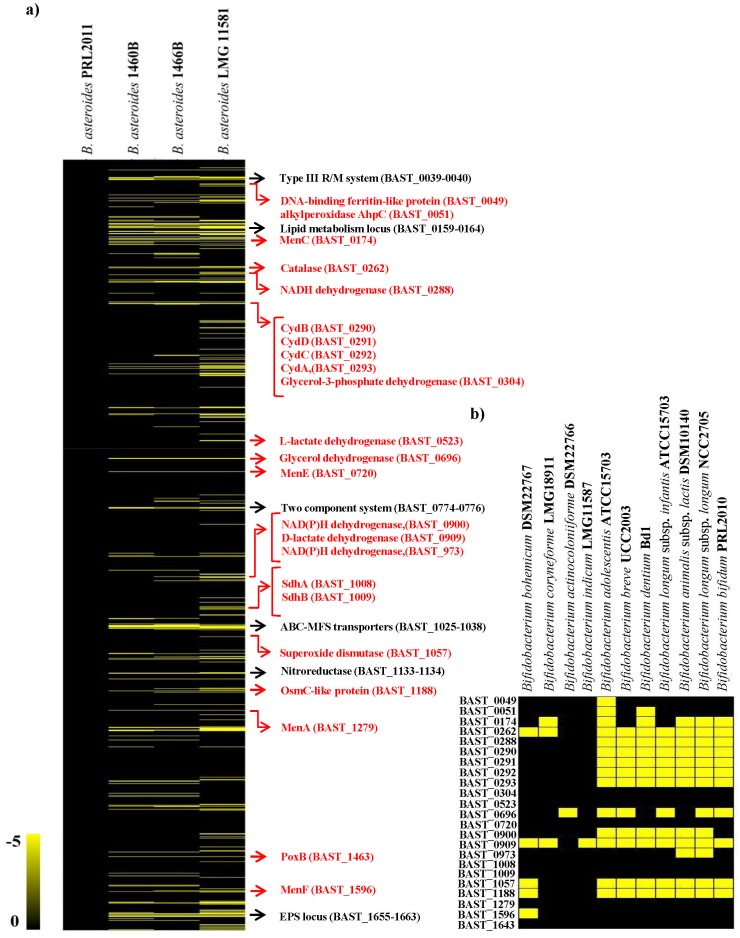
Genomic diversity in the *B. asteroides* phylogenetic group with reference to the *B. asteroides* strain PRL2011 genome. Panel a shows the comparative genomic hybridization data obtained using different members of the *B. asteroides* species. Each horizontal row corresponds to a probe on the array, and genes are ordered vertically according to their position on the PRL2011 genome. The columns represent the analysed strains, and strains are indicated by their strain codes. The colour code corresponding to the presence/absence is given at the top right of the figure: the gradient goes from black to yellow to indicate the presence, divergence or absence of a gene sequence. The predicted function of particular genes is shown on the right-hand margin. Black typed descriptions relate to most significant DNA regions that are absent in the investigated strains. Red typed descriptions represent DNA regions that encode enzymes predicted to be involved in respiration in PRL2011. Panel b details the presence (black) or absence (yellow) of key genes predicted to be involved in respiration within the *B. asteroides* phylogenetic group as well as in other genome sequenced bifidobacteria based on genomic data.

To determine the distribution of these presumed genetic determinants for respiration, the draft genome sequences of several other *Bifidobacterium* species isolated from insects, i.e. *Bifidobacterium bohemicum* DSM22767 [9], *Bifidobacterium coryneforme* LMG18911 [10], *Bifidobacterium actinocoloniiforme* DSM22766 [9] and *Bifidobacterium indicum* LMG11587 [10] (Bottacini et al., unpublished data), were used for comparative genomic analyses. Notably, apart from the catalase and superoxide dismutase-encoding genes which appear to be absent in these organisms, all other respiratory genes identified in this study were shown to be present within this set of genomes (Fig. 4). Furthermore, comparative Genome Hybridization (CGH) using *B. asteroides* PRL2011-based microarrays (Fig. 4) and involving three *B. asteroides* isolates revealed that the presumed respiratory genes are conserved within these three *B. asteroides* strains. The CGH data also revealed regions of diversity, most of which correspond to the predicted mobilome of PRL2011.

### Functional genomics analyses of *B. asteroides* PRL2011 and adaptation to putative respiratory metabolism

**Figure 5:**
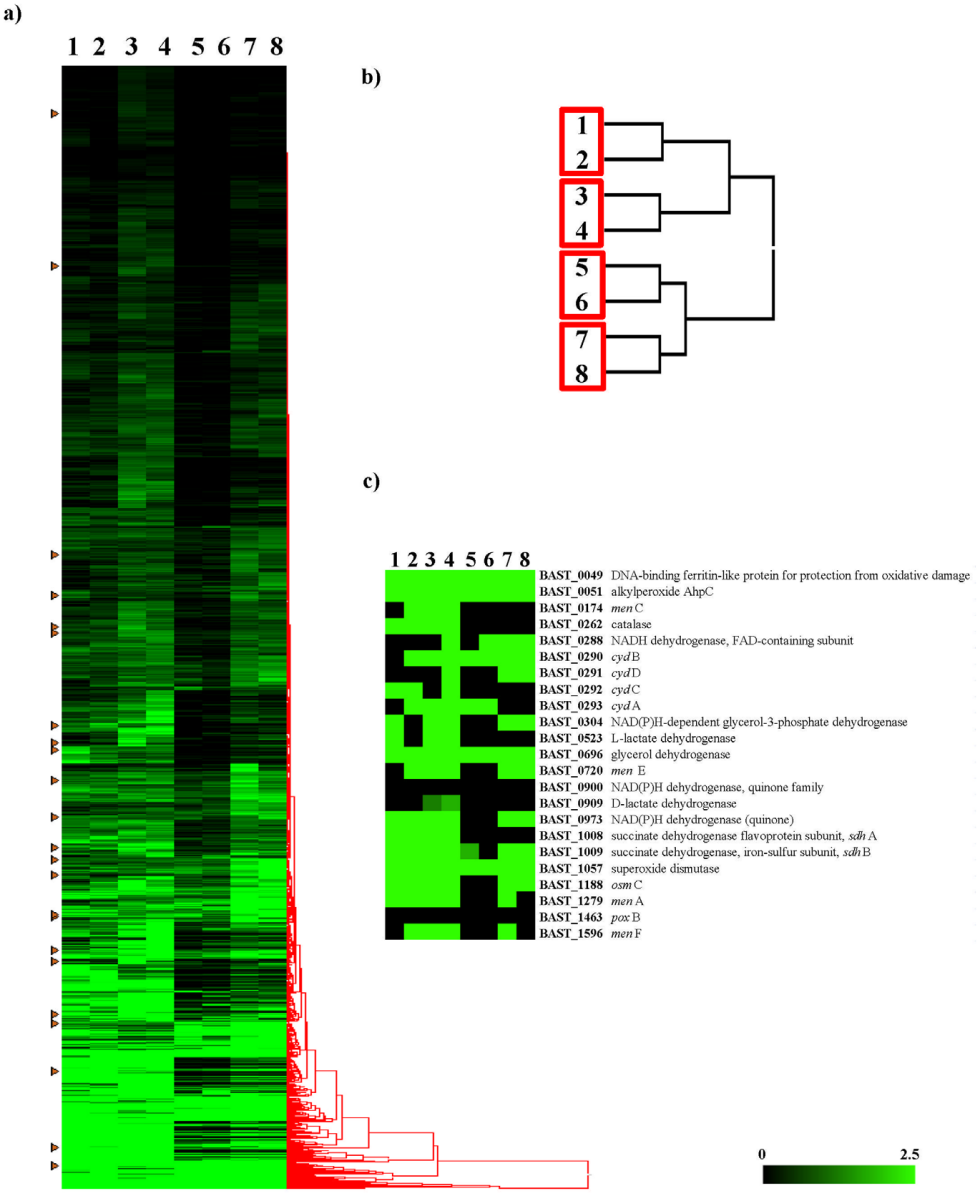
Identification of *B. asteroides* PRL2011 genes differentially expressed during growth in aerobic and anaerobic conditions. Panel a displays the global transcription profiling of PRL2011 cells under different growth conditions. Panel b shows the whole genome transcriptome-based clustering data analysis. Panel c represents a heat-map indicating the change in transcription levels of putative respiratory chain-encoding genes upon cultivation of PRL2011 cells under different cultivation conditions. Lane 1, transcriptome of PRL2011 cells cultivated in the absence of oxygen and protoporphyrin; lane 2, transcriptome of PRL2011 cells in the presence of oxygen and protoporphyrin; lane 3, transcriptome of PRL2011 cells in the absence of oxygen and hemin; lane 4, transcriptome of PRL2011 cells in the presence of oxygen and hemin; lane 5, transcriptome of PRL2011 cells in the absence of oxygen and glucuronic acid; lane 6, transcriptome of PRL2011 cells in the presence of oxygen and glucuronic acid; lane 7, transcriptome of PRL2011 cells in the presence of iron chloride (3 mM) and under anaerobic conditions; lane 8, transcriptome of PRL2011 cells in the presence of iron chloride (3 mM) and under aerobic conditions. Each row represents a separate transcript and each column represents a separate sample. Colour legend is on the bottom of the figure. Green indicates DNA regions which are actively transcribed, while black represents DNA regions that exhibit no or very low transcriptional activity. In panel a, the arrows indicate the change in the transcription of PRL2011 genes encoding for putative respiratory chain.

Global transcription profiling of PRL2011 cultivated in the presence of oxygen did not reveal any major expression changes respect to the transcriptome of PRL2011 grown under anaerobic conditions (Fig. 5). However, when the mRNA amounts were investigated for each identified gene of PRL2011 when cultivated under these conditions, it was noticed that many of the genes predicted to encode respiratory chain components were also transcribed in the absence of oxygen. A similar scenario was previously noticed for other respiring microorganisms such as *L. lactis* [55], [56] (Fig. 5). Crucial genes such as those encoding cytochrome oxidase were shown to be highly expressed in both aerobic and anaerobic conditions, which suggests that PRL2011 is constitutively expressing the enzymes involved in respiration or that a specific environmental signal is needed to (further) enhance the expression of these genes. The putative regulators *arc*A/B and FNR encoding genes of PRL2011 did not reveal any significant change in transcription levels when PRL2011 was cultivated under aerobic vs. anaerobic conditions, and together these findings may indicate that induction of the putative respiration network requires as yet unknown cultivation conditions or additional regulatory molecules.

Instead the genes encoding for ROS-protecting enzymes, such as the OsmC-like protein, did not reveal difference in its transcription level between anaerobic and aerobic conditions, thus indicating that it is constitutively expressed (Fig. 5).

Since respiration-directed ATP generation requires ATP synthase, we tested the ATPase activity of *B. asteroides* PRL2011 membranes, which was shown to be around 5-fold higher under aerobic relative to anaerobic conditions, regardless of the presence or absence of 10 µg/mL protoporphyrin IX (Fig. S8; Fig. S9). Different ATPase inhibitors were tested in order to identify the type of ATPase responsible for the observed (increased) activity. Neither ortho-vanadate, which specifically inhibits P-type ATPases, nor K_2_NO_3_, which targets V-type ATPases, were found to significantly affect the total ATPase activity (data not shown) [57], [58]. In contrast, DCCD significantly decreased ATPase activity, suggesting that F_1_F_0_ ATPases are responsible for around the 32–38% of the total ATPase activity in *B. asteroides* membranes.

Interestingly, F_1_F_0_-ATPase activity represented 57–63% of the total activity when protoporphyrin IX was included in the growth media, whereas in the absence of protoporphyrin IX the specific F_1_F_0_-ATPase activity was significantly lower, representing approximately 38% of the total membrane ATPase activity, as deduced from the values presented in Figure S8. This suggests that the presence of protoporphyrin IX, a heme precursor [59], promotes an increase in the ATPase activity of the membrane-bound enzymatic complex IV.

In previous work, we observed that F_1_F_0_ ATPase was responsible for most of the ATPase activity found in membrane-containing fractions of two strains of *B. animalis* subsp. *lactis* [60]. It is known that in other bifidobacteria lacking a functional respiratory chain, this enzyme is crucial for the maintenance of the internal pH [61].

## Conclusions

Bifidobacteria have for long been considered anaerobic or microaerophilic microorganisms, without any indication that these micro organisms may harbor any capabilities for respiratory metabolism [51]. Genome analysis of *B. asteroides* predicted the presence of a respiratory chain that may allow aerobic respiration by these bacteria. Our findings suggest that *B. asteroides* represent the first reported case of such a metabolic ability within the genus *Bifidobacterium*. Among members of the *Actinobacteria* phylum a respiratory pathway has been identified in representatives of the CMN group, which includes the genera *Corynebacterium*, *Mycobacterium* and *Nocardia* [51], [62]. Here, we have provided solid genetic and physiological evidence that *B. asteroides* PRL2011 is adapted to being exposed to oxygenated environments, and may avail of oxygen by adjusting its metabolism, which is consistent also with its ecological niche, but that this apparently requires specific growth conditions that have not yet been defined. Notably, phylogenomics-based analyses revealed the distant relationship between *B. asteroides* and other bifidobacterial species, thus reinforcing previous findings about the evolutionary development of this species [63].

One may thus argue that the respiratory gene set is present in *B. asteroides* to allow adaptation to the aerated insect gut, whereas this property was lost or not acquired in bifidobacteria that inhabit the anaerobic gut of mammals. Furthermore, the gene set involved in the putative respiration metabolism do not display any characteristic evidence of recent HGT acquisition (e.g., atypical codon usage and/or deviation from GC content). In addition, from an evolutionary prospective it is interesting to underline that insects roamed the earth well before mammals did, and one may thus argue that insects may have been the original host of bifidobacteria. Apart from the putative respiratory metabolism, the presence of *B. asteroides* PRL2011 genes specifying bacterial structures that are known to mediate the interaction with the host in other bifidobacteria, such as capsular polysaccharides and type IVb pili [1], [30], [64], may represent genetic signatures of an evolutionary development that lead to the bifidobacterial adaptation to colonize the mammalian gut. The predicted ability of *B. asteroides* to grow in the presence of oxygen has important implications for the interaction of this bacterial species with its oxygenated hosts. With respect to its role as a member of the hindgut microbiota of bees [11], [12], [13], the ability to tolerate and metabolically exploit O_2_ may account for the observation that *B. asteroides* is frequently found in high numbers in this particular ecological niche.

Another intriguing finding that supports the ability of *B. asteroides* PRL2011 to utilize oxygen is the presence in its genome of genes encoding enzymes such as a catalase and superoxide dismutase, which are involved in the protection against the negative effects produced by oxygen in an aerobic environment [65].

The present study represents a firm basis from which we can further define the factors that shape microbial ecology in a particular environment such as the insect's intestine, which may represent a valuable model to investigate intricate microbe-microbe as well as microbe-host relationships within complex ecosystems such as that of the mammalian gut.

## Materials and Methods

### Bacterial strains, growth conditions and chromosomal DNA extraction

Cultures were cultivated anaerobically/aerobically in MRS (Sharlau, Barcelona, Spain) and incubated at 37°C for 16 h. Bacterial DNA was extracted as described previously [66] and subjected to further phenol/chloroform purification using the protocol described previously [67].

### Carbohydrate growth assay

Cell growth on semi synthetic MRS medium supplemented with 1% (wt/vol) of a particular sugar was monitored by optical density at 600 nm using a plate reader (Biotek, Vermont, USA). The plate reader was run in discontinuous mode, with absorbance readings performed in 60 min intervals, and preceded by 30 sec shaking at medium speed. Cultures were grown in biologically independent triplicates and the resulting growth data were expressed as the mean of these replicates. Carbohydrates were purchased from Sigma and Carbosynth (Berkshire, UK).

### Genome sequencing and assembly

Chromosomal DNA was mechanically sheared via a Hydroshear device (Genemachine, San Carlos, CA), the prepared inserts were then ligated into appropriate vectors. A two-fold fosmid library was constructed using the CopyControl™ Fosmid Production Kit (Epicentre). DNA was sheared and fragment size selected on an agarose pulsed-field gel electrophoresis, excised and purified before ligation in the pCCqFos vector. A 30-fold sequencing coverage using pyrosequencing technology on a 454 FLX instruments was used. The files generated by the 454 FLX instrument were assembled with Newbler software to generate a consensus sequence, which was then used for assembly using data from Sanger sequencing of the Fosmid library using the Arachne genome assembly software. Two rounds of additional sequencing walks were performed resulting in a single contig (2,167,301 bp). Quality improvement of the genome sequence involved sequencing of over 500 PCR products (3000 sequencing reads) across the entire genome to ensure correct assembly, double stranding and the resolution of any remaining base-conflicts. The genome sequence was finally edited to a Phred confidence value of 30 or more. Based on the final consensus quality scores, we estimate an overall error rate of less than 1 error per 10^5^ nucleotides.

### Sequence annotation

Protein-encoding open reading frames (ORFs) were predicted using a combination of BLASTX [68] and Prodigal [69]. Results of these gene finder programs were combined manually and a preliminary identification of ORFs was performed on the basis of BLASTP [68] analysis against a non-redundant protein database provided by the National Centre for Biotechnology Information. Artemis [70] was employed to inspect the results of the combined gene finders and its associated BLASTP results, which was used for a manual editing effort in order to verify, and if necessary, redefine the start of every predicted coding region, or to remove or add coding regions.

Assignment of protein function to predicted coding regions of *B. asteroides* PRL2011 genome was performed manually. Moreover, the revised gene/protein set was searched against the Swiss-Prot/TrEMBL, PRIAM, protein family (Pfam), TIGRFam, Interpro, KEGG, and COGs databases, in addition to BLASTP vs. nr. From all these results, functional assignments were made. Manual corrections to automated functional assignments were completed on an individual gene-by-gene basis as needed.

### Bioinformatic analyses

Transfer RNA genes were identified using tRNAscan-SE [71]. Ribosomal RNA genes were detected on the basis of BLASTN searches and annotated manually. Insertion sequence elements were identified using Repeat-finder [72], and BLAST [68] and annotated manually. IS families were assigned using ISFinder (http://www-is.biotoul.fr/is.html). Carbohydrate-active enzymes were identified based on similarity to the carbohydrate-active enzyme (CAZy) database entries [73] and transporter classification was performed according to the TCDB scheme [74].

Variances in GC content were profiled by the DNA segmentation algorithm hosted at http://tubic.tju.edu.cn/GC-Profile/ [75], atypical codon usage regions were mapped using the factorial correspondence analysis through the assistance of the GCUA software.

### Oxygen consumption

Evaluation of oxygen consumption was performed using *B. asteroides* PRL2011 cells collected at early stationary phase (OD600 value of 1). Briefly, PRL2011 cells were harvested by centrifugation 3000 rpm for 5 min and resuspended in 1 ml of water. Then, 100 µl bacterial cell suspension was placed in the electrode chamber of an oxygraph instrument (Hansatech Instrument) where oxygen consumption was monitored for 5 min.

### Hydrogen peroxide resistance level of PRL2011

Tolerance to H_2_O_2_ exposure of PRL2011 strain was evaluated by cultivating cells in presence of hydrogen peroxide at a concentration of 0.002% or 0.004%, which were previously shown to be inhibitory for bifidobacteria [19]. Growth was monitored using a microplate reader (Biotek, Vermont, USA), which have been set as described above. Furthermore, tolerance of PRL2011 to hydrogen peroxide was monitored by exposing an exponential culture to 0.002% or 0.004% H_2_O_2_ for 2 h, after which survival was then evaluated by viable count determination on MRS agar (incubation at 37°C for 16 h; Sharlau, Barcelona, Spain).

### CGH microarray data acquisition and treatment

Fluorescence scanning was performed on an Innoscan confocal laser scanner (Innopsis, France). Signal intensities for each spot were determined using Microarray Imager 5.8 software (CombiMatrix, Mulkiteo, USA). Signal background was calculated as the mean of negative controls plus 2 times the standard deviation [76]. A global quantile normalization was performed [77] and log2 ratios among the reference sample (*B. asteroides* PRL2011) and the other samples analyzed were calculated. The distribution of the log2-transformed ratios was calculated for each hybridization reaction separately. Log2-transformed ratios of each probe were visualized and ranked by position on the *B. asteroides* PRL2011 genome by a heatmap using TMev 4.0 software (http://www.tm4.org/mev.html). Hierarchical clustering was performed with average linkage and euclidean distance [78] using TMev 4.0 software.

### RNA isolation

RNA was isolated according to the protocol described previously [79]. The quality of the RNA was checked by analysing the integrity of rRNA molecules by Experion (BioRad).

### Expression microarray and data analysis

Microarray analysis was performed with a *B. asteroides* PRL2011-based array. A total of 6432 probes, representing 1680 ORFs of the *B. asteroides* PRL2011 genome, of 35–40 bp in length were designed using OligoArray 2.1 software [80]. Oligos were synthesized in 4 replicates on a 2×40 k CombiMatrix array (CombiMatrix, Mulkiteo, USA). Replicates were distributed on the chip at random, non-adjacent positions. A set of 29 negative control probes designed on phage and plant sequences were also included on the chip in 60 replicates at randomly distributed positions.

Reverse transcription and amplification of 500 ng of total RNA was performed with MessageAmp II-Bacteria kit (Ambion, Austin TX) according to the manufacturer's instructions. 5 µg of mRNA was then labelled with ULS Labelling kit for Combimatrix arrays with Cy5 (Kreatech, The Netherlands). Hybridization of labeled cDNA to *B. asteroides* PRL2011 arrays was performed according to CombiMatrix protocols (http://www.combimatrix.com/support_docs.htm).

Following hybridization, microarrays were washed as described in the manual and scanned using Innoscan confocal laser scanner (Innopsis, France). A gene was considered differentially expressed between a test condition and a control when an expression ratio of between 0.2 and 5relative to the result for the control was obtained with a corresponding P value that was less than 0.001. Final data presented are the averages from at least two independent array experiments.

### Analyses of enzymatic activities

Specific ATPase activity was calculated from the measurement of the inorganic phosphate released using the malachite green assay [60]. Inside-out membrane vesicles, obtained from cells grown in the absence/presence of 10 µg/mL protoporphyrin IX (Sigma-Aldrich), and under aerobic or anaerobic conditions, were prepared as previously described [60]. Five µg of membrane protein were incubated in the absence/presence of the following ATPase inhibitors: 0.2 mM N,N′-dicyclohexylcarbodiimide (DCCD) (Sigma-Aldrich), 0.2 mM ortho-vanadate (Sigma-Aldrich) or 25 mM K_2_NO_3_ for 10 min at 37°C, and subsequently for 60 min in ice in 50 mM MES-potassium buffer (pH 5.25), containing 5 mM MgCl_2_ ATP (disodium salt, GE Healthcare) was added at a final concentration of 300 µM to initiate the reaction, which was performed in a final volume of 100 µl, and that was developed during 10 min at 37°C. Finally, the reaction was stopped by the addition of 100 µl of malachite green solution, containing 0.03% (w/v) malachite green, 0.2% (w/v) sodium molybdate and 0.05% (w/v) Triton X-100 in HCl 0.7 M. The Abs_650_ of the reaction was read after 10 min, and compared with the values of a standard curve made with inorganic phosphate (KH_2_PO_4_). In our experiments, one unit of ATPase activity was defined as the amount of enzyme that catalyzed the release of 1 µmol of inorganic phosphate in one min.

## Supporting Information

Figure S1 **Glycobiome analysis of** ***B. asteroides*** **PRL2011.** Panel a displays growth curves of *B. asteroides* PRL2011 in a growth medium containing varying carbohydrates as a sole carbon source (panel a). Panels b, c and d show the glycoside-hydrolase (GH) families, glycosyltransferases (GT) families and carbohydrate esterases (CE) families identified in the genome of *B. asteroides* PRL2011 according to the CAZy database [75], respectively, compared to other bacteria.
(TIF)

Figure S2 **Genetic analyses of** ***nad*** **H locus in** ***B. asteroides*** **PRL2011.** Panels a–c shows the comparison of the *nad*H locus in *B. asteroides* PRL2011 with the corresponding loci in different bacteria. Panel d–f represents the phylogenetic tree based on the NADH dehydrogenase. Each arrow indicates an ORF. The length of the arrow is proportional to the length of the predicted ORF. Corresponding genes are marked with the same colour. Putative function of the protein is indicated above each arrow.
(TIF)

Figure S3 **Genomic analysis of the** ***cyd*** **locus in** ***B. asteroides*** **PRL2011.** Panel a displays the comparison of the *cyd* locus in *B. asteroides* PRL2011 with the corresponding loci in different bacteria. Panel b shows the phylogenetic supertree obtained by the concatenation of the gene encompassing the *cyd* operon. Each arrow indicates an ORF. The length of the arrow is proportional to the length of the predicted ORF. Corresponding genes are marked with the same colour. Putative function of the protein is indicated above each arrow.
(TIF)

Figure S4 **Additional respiratory-related gene clusters.** Genes are represented by arrow and are coloured according to predicted function indicated in the figure.
(TIF)

Figure S5 **Mobile genetic elements of the** ***B. asteroides*** **PRL2011 genome.** The first plot from the top shows the most significant regions putatively acquired by HGT, IS elements are labelled in red, regions 1 and 4 indicates the R/M systems, region 2 depicts the EPS locus, region 3 represents the cluster involved in lipid metabolism. In the second plot, all the genes of *B. asteroides* PRL2011 supposed to be acquired by HGT. In the third plot each dot represents an ORF displaying a biased codon usage determined by factorial correspondence analysis of codon usage. The fourth plot indicates the deviation of the G+C content of those ORFs whose G+C content values is higher or less the average G+C values± standard deviation of the *B. asteroides* PRL2011 genome from the mean average (60.49%).
(TIF)

Figure S6 **Comparison of the** ***mle*** **E locus in** ***B. asteroides*** **PRL2011 with the corresponding loci in different bacteria.** Each arrow indicates an ORF. The length of the arrow is proportional to the length of the predicted ORF. Corresponding genes are marked with the same colour. The putative function of corresponding proteins is indicated above each arrow.
(TIF)

Figure S7 **RCC1 proteins identified in** ***B. asteroides*** **PRL2011 genome.** Panel a represents alignments of repeats from RCC1 proteins of *B. asteroides* PRL2011. RCC1-, LPTGX- and *Listeria*-*Bacteroidetes* domains are indicated. Panel b displays phylogenetic tree analysis of RCC1 proteins.
(TIF)

Figure S8 **Evaluation of the ATPase activity of** ***B. asteroides*** **PRL2011.** Panel a and b represents the ATPase activity in membrane vesicles of *B. asteroides* PRL2011 grown in the absence or presence of 10 µg/mL protoporphyrin IX or hemin, respectively,and in anaerobiosis or aerobiosis. The F_1_F_0_ ATPase activity was calculated as the difference between the total ATPase activity and the ATPase activity measured in experiments containing the specific inhibitor DCCD. Error bars represent standard deviations experiments with three different batches of membrane vesicles. The activity is expressed as units per mg of protein.
(TIF)

Figure S9 **Evaluation of viability of** ***B. asteroides*** **PRL2011 in presence of hydrogen peroxide and survival rate of PRL2011 upon storage at 4°C.** Panel a indicates the growth of *B. asteroides* PRL2011 as well as the enteric *B. bifidum* PRL2010 and *B. animalis* subsp. *lactis* DSM10140 cultivated in the presence of 0.0036% hydrogen peroxide (dotted lines) compared to the same strains cultivated in absence of hydrogen peroxide (solid lines). Panel b displays the rate of survival of *B. asteroides* PRL2011 as well as the enteric *B. bifidum* PRL2010 and *B. animalis* subsp. *lactis* DSM10140 exposed to 0.0036% hydrogen peroxide for 1.5 h (pale grey bar) and without hydrogen peroxide (dark grey bar). Panel c depicts viability of PRL2011 cultures in the presence or absence of oxygen upon storage at 4°C.
(TIF)

Table S1 **Genome features of** ***B. asteroides*** **PRL2011.**
(DOC)

Table S2 **ORFs identified on the** ***B. asteroides*** **PRL2011 genome which have no significant homology to other currently available bifidobacterial ORFs.**
(DOC)

Table S3 **Predicted secretome of** ***B. asteroides*** **PRL2011.**
(DOC)

Text S1 **The mobilome of** ***B. asteroides*** **genome, the secretome of** ***B. asteroides*** **PRL2011, stability of** ***B. asteroides*** **cultures in presence of hydrogen peroxide, evaluation of growth survival of** ***B. asteroides*** **cultures over time.**
(DOC)
