# Supplementary figures and images for: Bifidobacterium asteroides PRL2011 Genome Analysis Reveals Clues for Colonization of the Insect Gut
Source: PLoS One. 2012 Sep 20;7(9):e44229. doi: 10.1371/journal.pone.0044229 (PMC3447821; doi:10.1371/journal.pone.0044229)

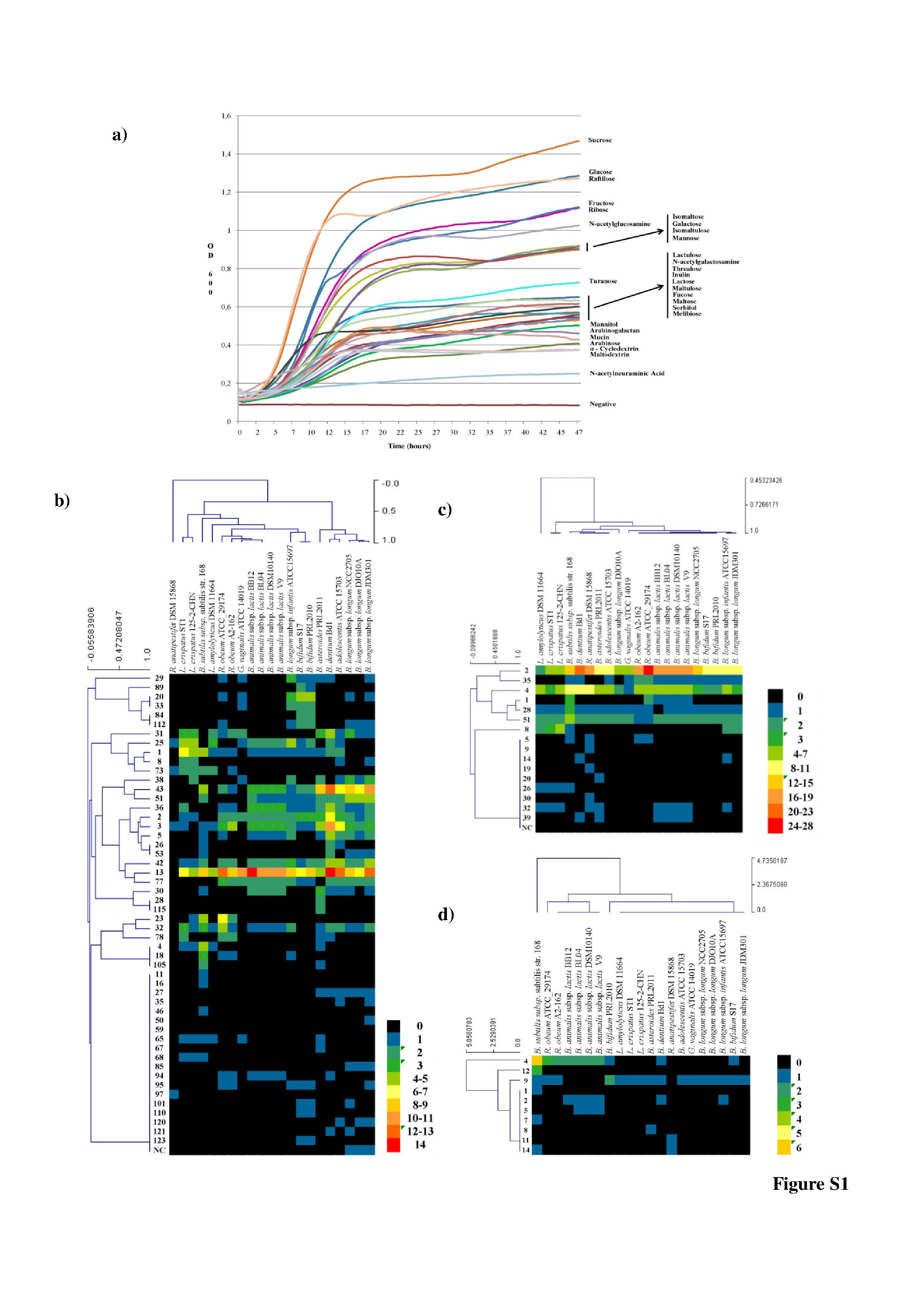

Supplement: Figure S1 — Glycobiome analysis of B. asteroides PRL2011. Panel a displays growth curves of B. asteroides PRL2011 in a growth medium containing varying carbohydrates as a sole carbon source (panel a). Panels b, c and d show the glycoside-hydrolase (GH) families, glycosyltransferases (GT) families and carbohydrate esterases (CE) families identified in the genome of B. asteroides PRL2011 according to the CAZy database [75], respectively, compared to other bacteria. (TIF) [file pone.0044229.s001.tif]

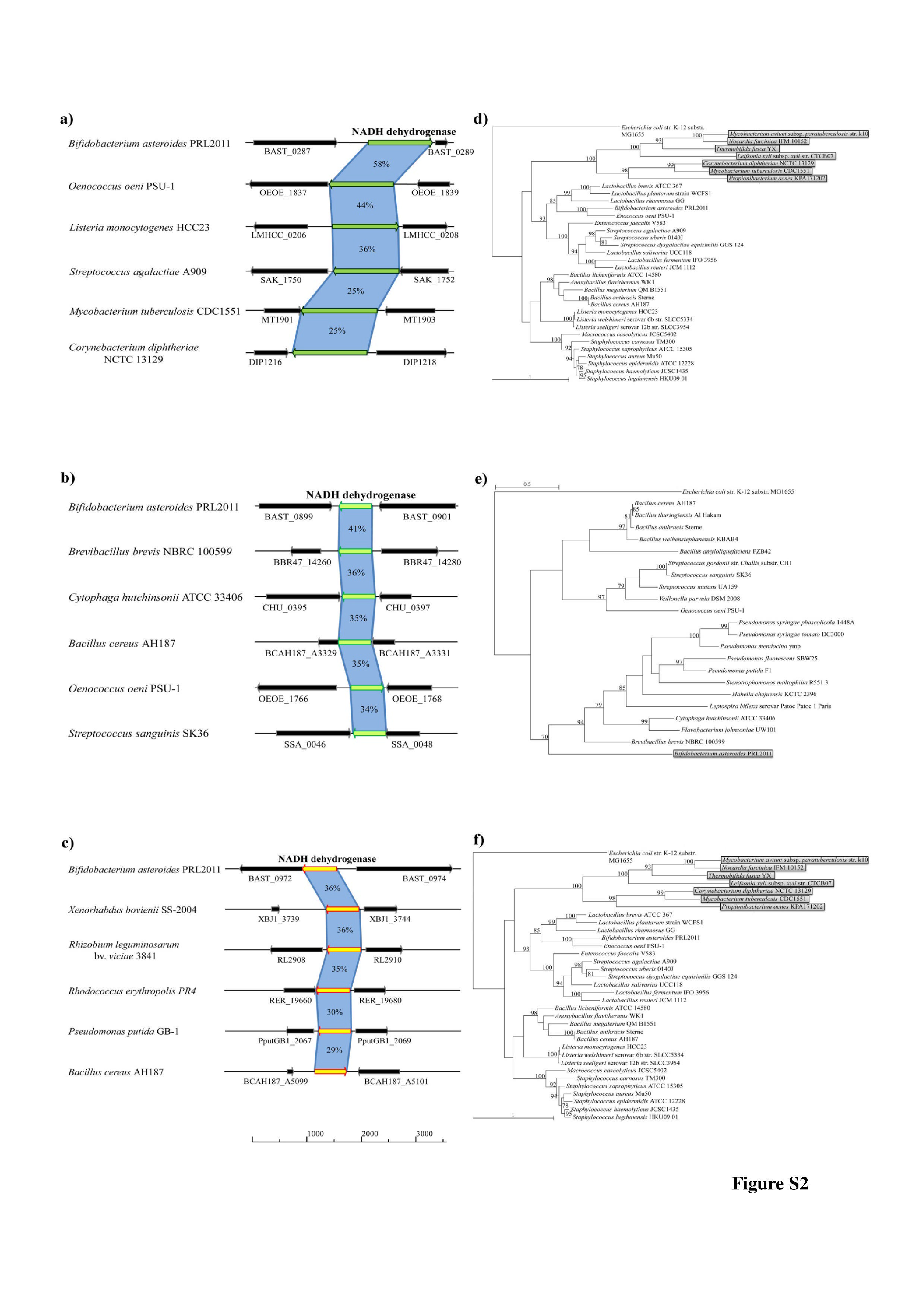

Supplement: Figure S2 — Genetic analyses of nad H locus in B. asteroides PRL2011. Panels a–c shows the comparison of the nadH locus in B. asteroides PRL2011 with the corresponding loci in different bacteria. Panel d–f represents the phylogenetic tree based on the NADH dehydrogenase. Each arrow indicates an ORF. The length of the arrow is proportional to the length of the predicted ORF. Corresponding genes are marked with the same colour. Putative function of the protein is indicated above each arrow. (TIF) [file pone.0044229.s002.tif]

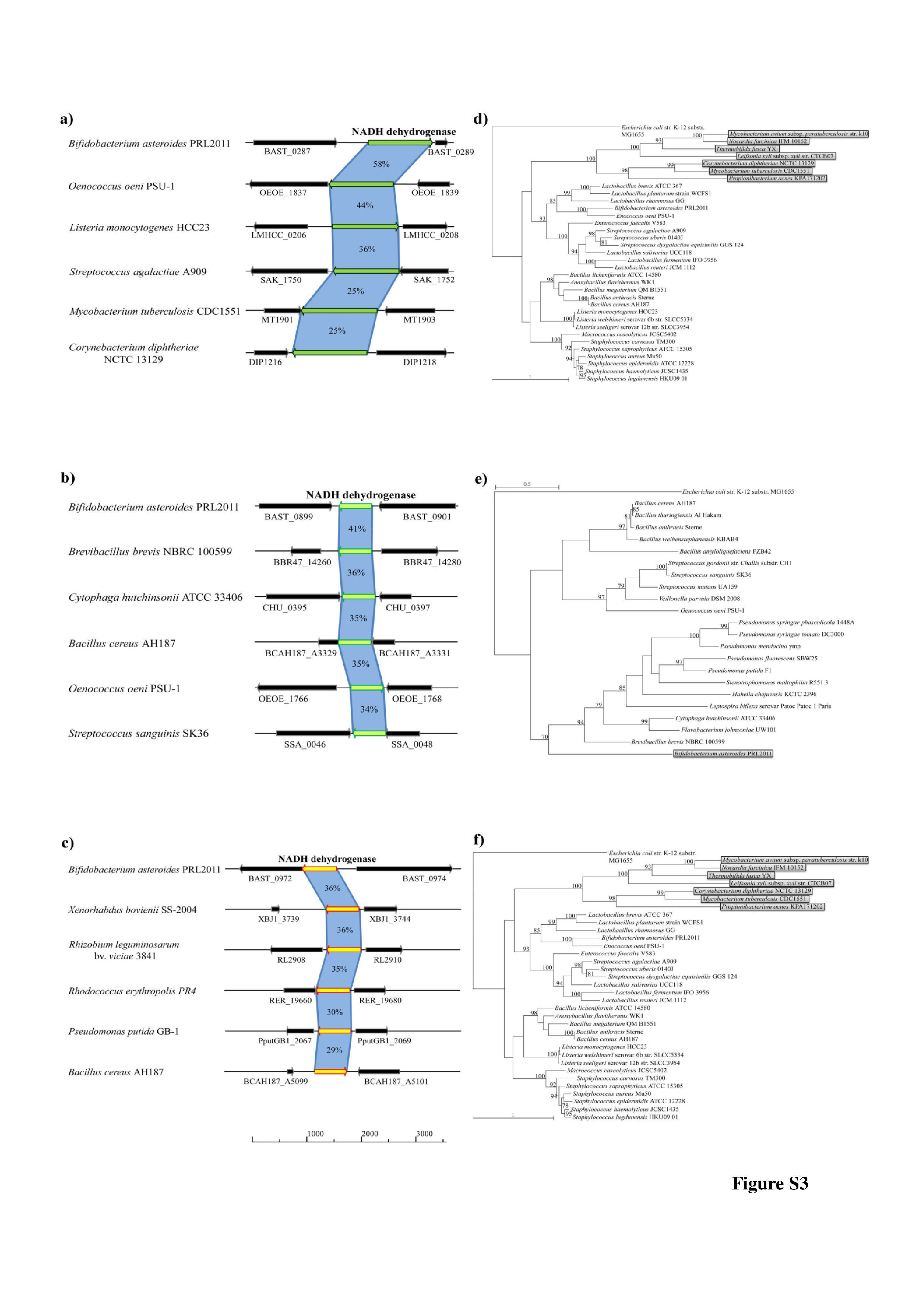

Supplement: Figure S3 — Genomic analysis of the cyd locus in B. asteroides PRL2011. Panel a displays the comparison of the cyd locus in B. asteroides PRL2011 with the corresponding loci in different bacteria. Panel b shows the phylogenetic supertree obtained by the concatenation of the gene encompassing the cyd operon. Each arrow indicates an ORF. The length of the arrow is proportional to the length of the predicted ORF. Corresponding genes are marked with the same colour. Putative function of the protein is indicated above each arrow. (TIF) [file pone.0044229.s003.tif]

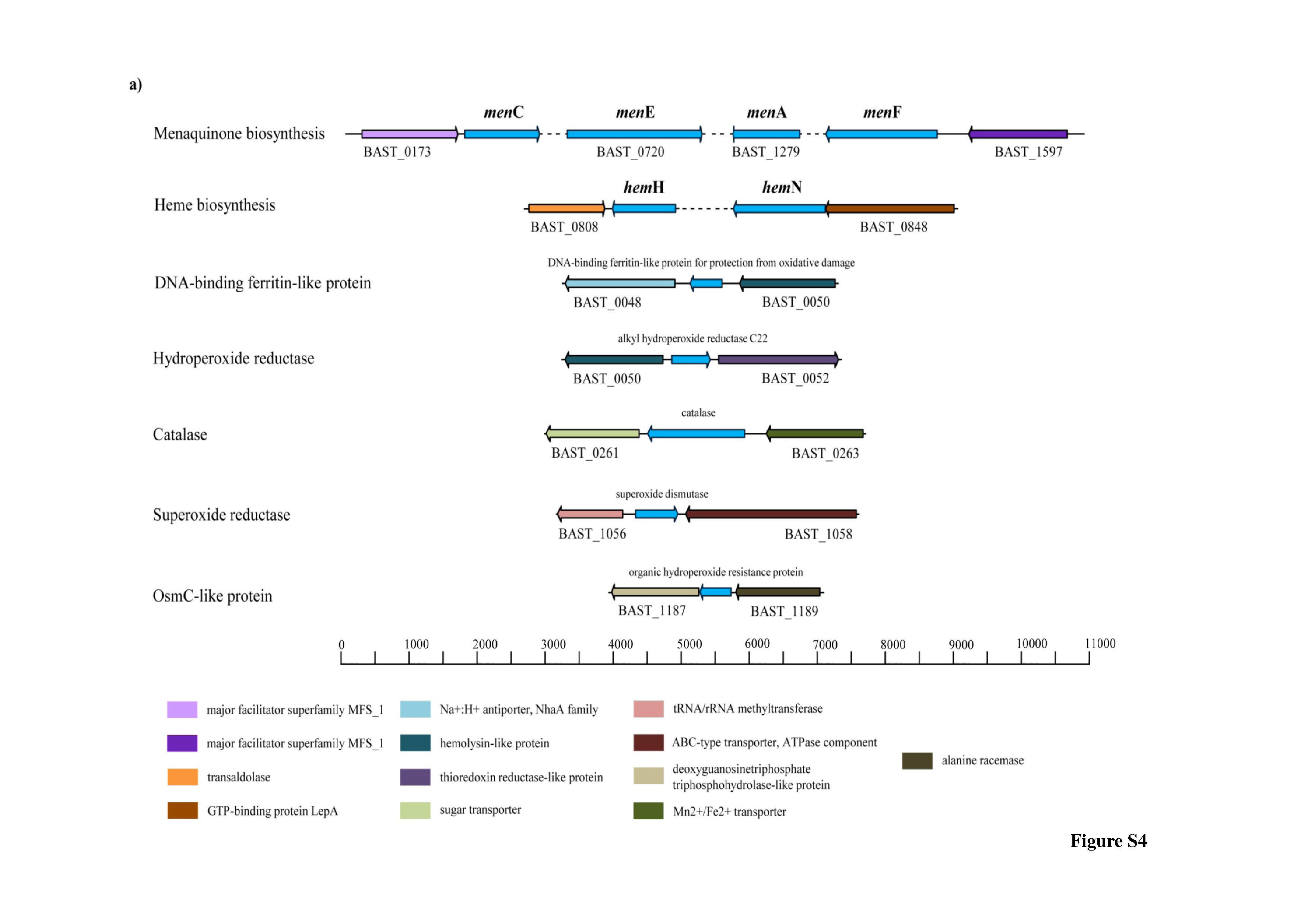

Supplement: Figure S4 — Additional respiratory-related gene clusters. Genes are represented by arrow and are coloured according to predicted function indicated in the figure. (TIF) [file pone.0044229.s004.tif]

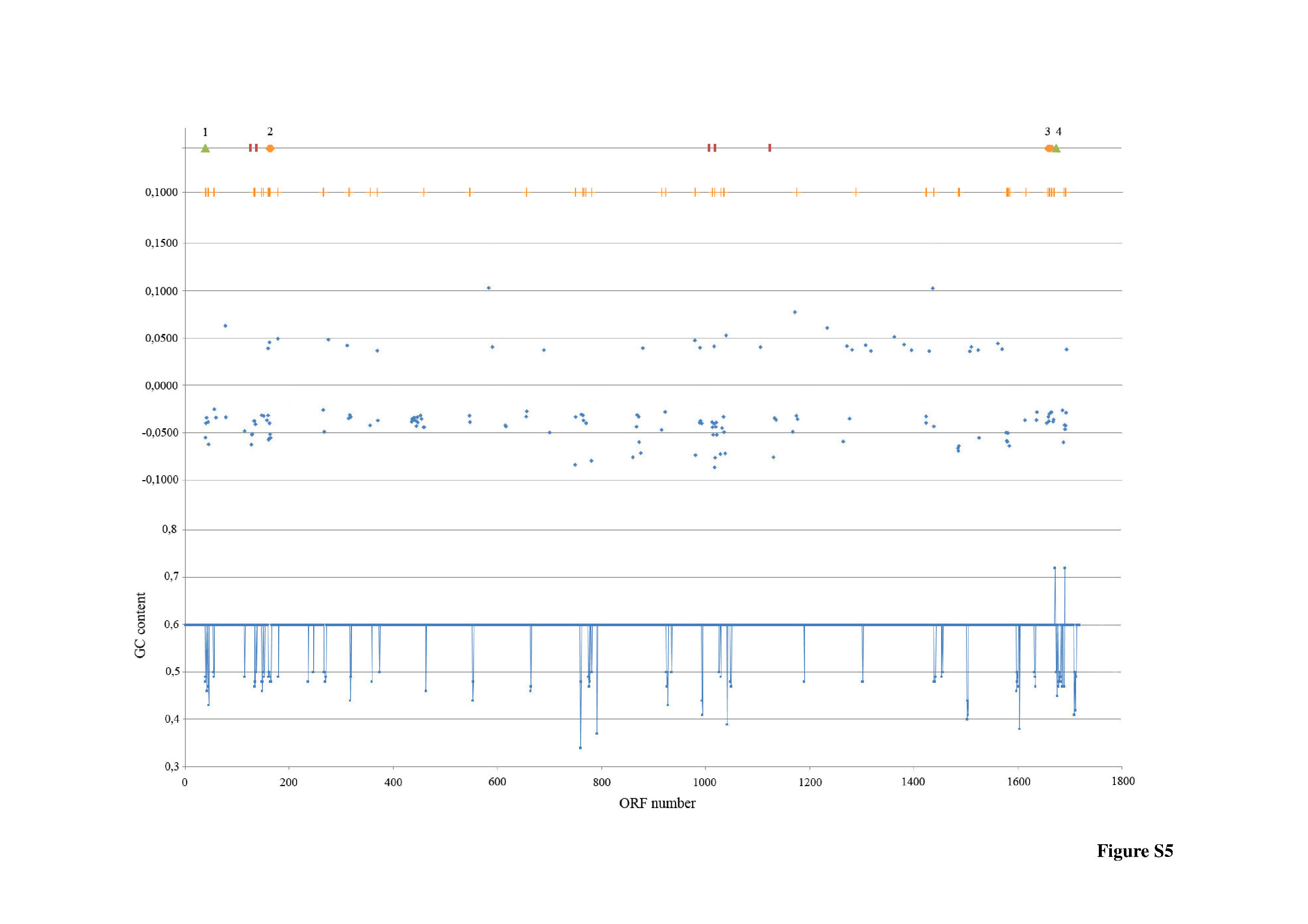

Supplement: Figure S5 — Mobile genetic elements of the B. asteroides PRL2011 genome. The first plot from the top shows the most significant regions putatively acquired by HGT, IS elements are labelled in red, regions 1 and 4 indicates the R/M systems, region 2 depicts the EPS locus, region 3 represents the cluster involved in lipid metabolism. In the second plot, all the genes of B. asteroides PRL2011 supposed to be acquired by HGT. In the third plot each dot represents an ORF displaying a biased codon usage determined by factorial correspondence analysis of codon usage. The fourth plot indicates the deviation of the G+C content of those ORFs whose G+C content values is higher or less the average G+C values± standard deviation of the B. asteroides PRL2011 genome from the mean average (60.49%). (TIF) [file pone.0044229.s005.tif]

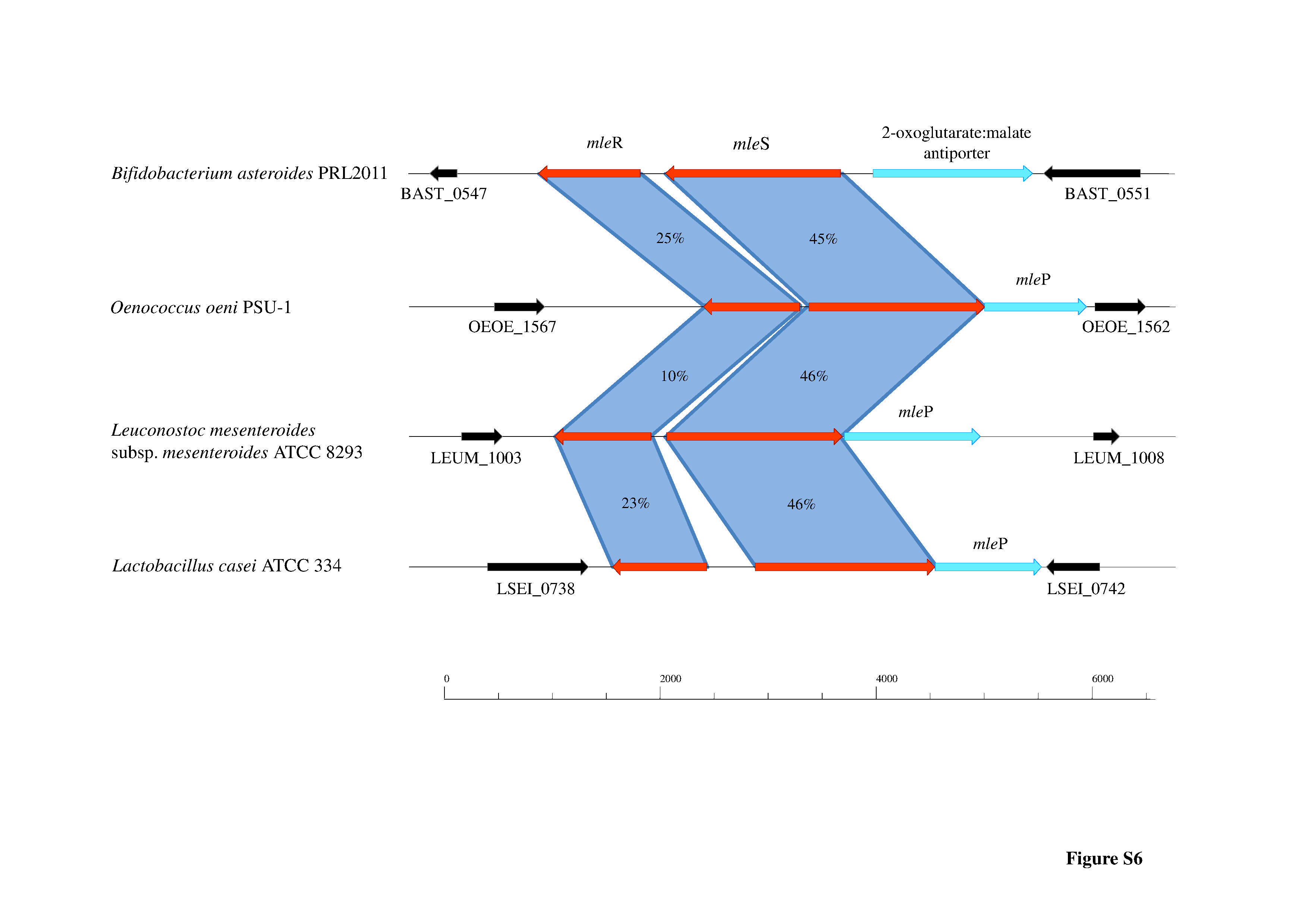

Supplement: Figure S6 — Comparison of the mle E locus in B. asteroides PRL2011 with the corresponding loci in different bacteria. Each arrow indicates an ORF. The length of the arrow is proportional to the length of the predicted ORF. Corresponding genes are marked with the same colour. The putative function of corresponding proteins is indicated above each arrow. (TIF) [file pone.0044229.s006.tif]

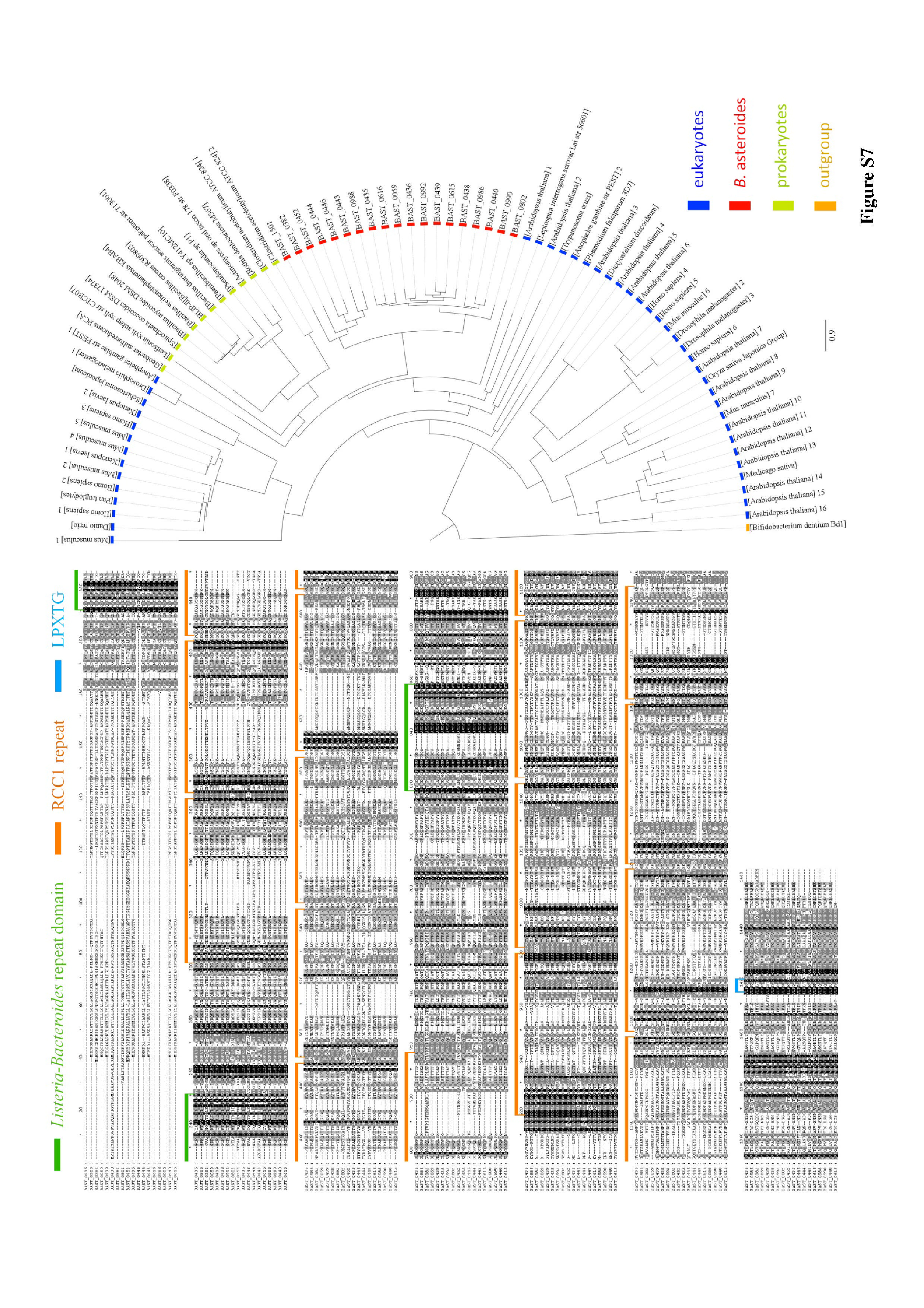

Supplement: Figure S7 — RCC1 proteins identified in B. asteroides PRL2011 genome. Panel a represents alignments of repeats from RCC1 proteins of B. asteroides PRL2011. RCC1-, LPTGX- and Listeria-Bacteroidetes domains are indicated. Panel b displays phylogenetic tree analysis of RCC1 proteins. (TIF) [file pone.0044229.s007.tif]

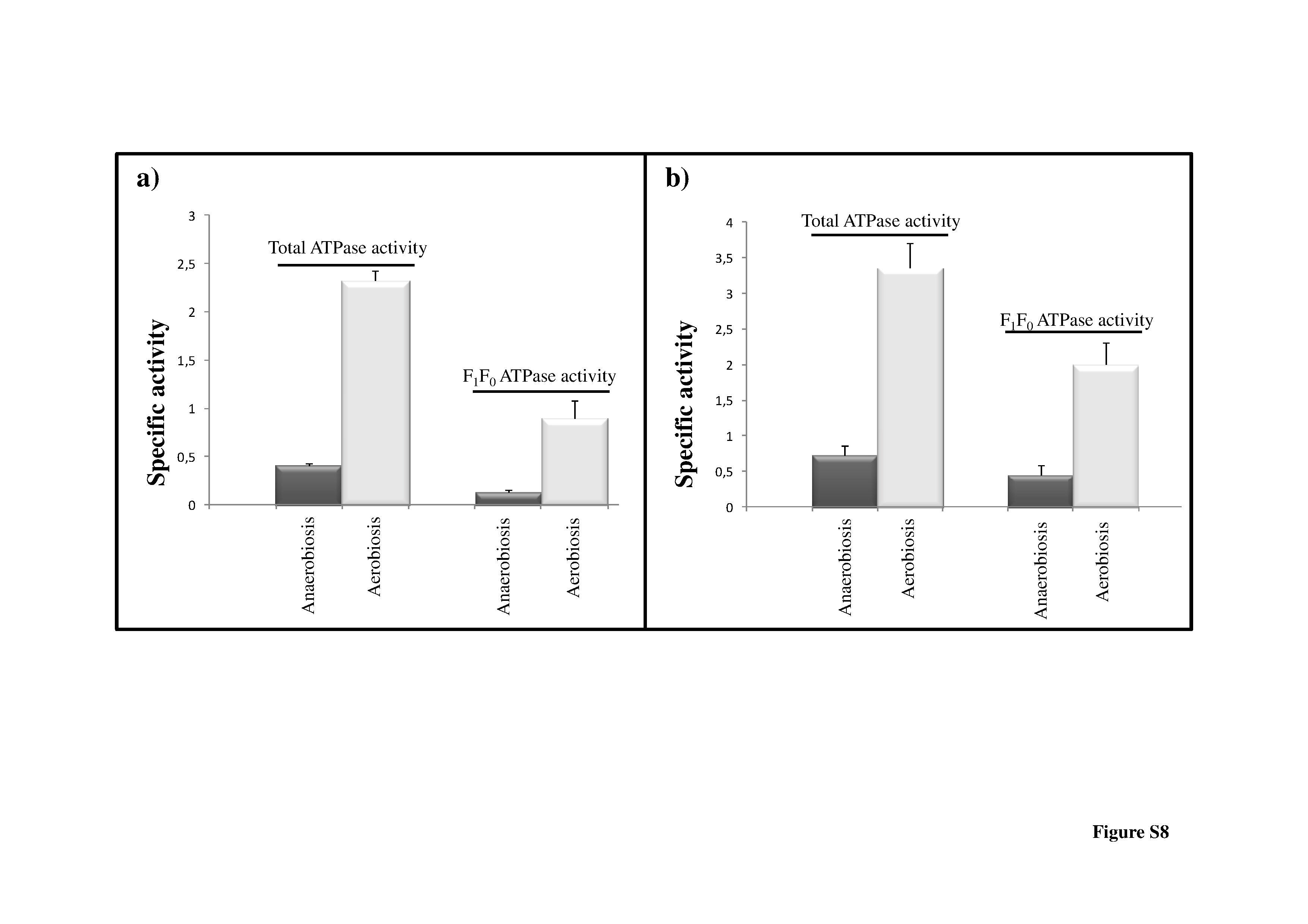

Supplement: Figure S8 — Evaluation of the ATPase activity of B. asteroides PRL2011. Panel a and b represents the ATPase activity in membrane vesicles of B. asteroides PRL2011 grown in the absence or presence of 10 µg/mL protoporphyrin IX or hemin, respectively,and in anaerobiosis or aerobiosis. The F1F0 ATPase activity was calculated as the difference between the total ATPase activity and the ATPase activity measured in experiments containing the specific inhibitor DCCD. Error bars represent standard deviations experiments with three different batches of membrane vesicles. The activity is expressed as units per mg of protein. (TIF) [file pone.0044229.s008.tif]

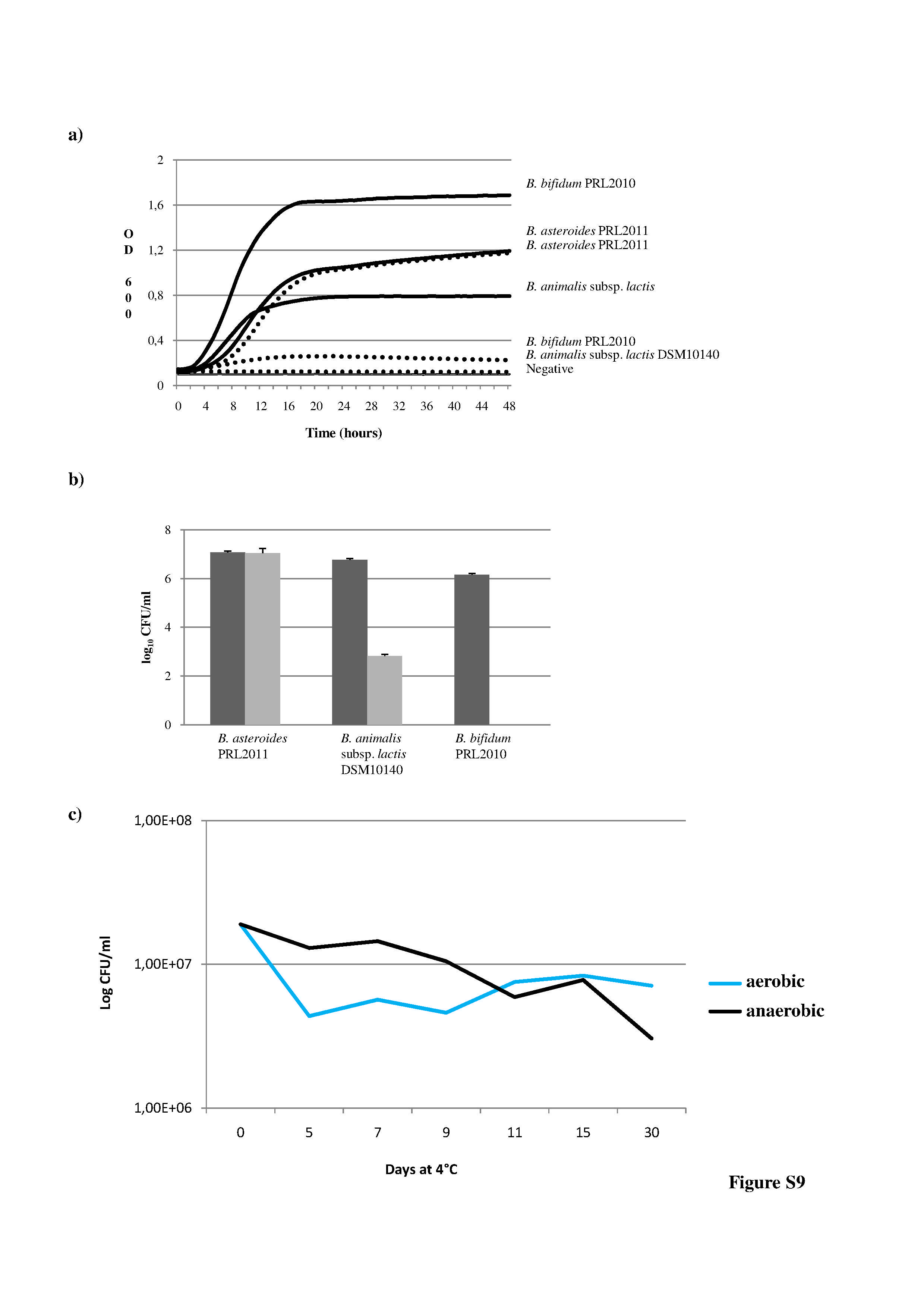

Supplement: Figure S9 — Evaluation of viability of B. asteroides PRL2011 in presence of hydrogen peroxide and survival rate of PRL2011 upon storage at 4°C. Panel a indicates the growth of B. asteroides PRL2011 as well as the enteric B. bifidum PRL2010 and B. animalis subsp. lactis DSM10140 cultivated in the presence of 0.0036% hydrogen peroxide (dotted lines) compared to the same strains cultivated in absence of hydrogen peroxide (solid lines). Panel b displays the rate of survival of B. asteroides PRL2011 as well as the enteric B. bifidum PRL2010 and B. animalis subsp. lactis DSM10140 exposed to 0.0036% hydrogen peroxide for 1.5 h (pale grey bar) and without hydrogen peroxide (dark grey bar). Panel c depicts viability of PRL2011 cultures in the presence or absence of oxygen upon storage at 4°C. (TIF) [file pone.0044229.s009.tif]
