# Supplementary material for: Bifidobacterium asteroides PRL2011 Genome Analysis Reveals Clues for Colonization of the Insect Gut
Source: PLoS One. 2012 Sep 20;7(9):e44229. doi: 10.1371/journal.pone.0044229 (PMC3447821; doi:10.1371/journal.pone.0044229)
Supplement: Table S1 — Genome features of B. asteroides PRL2011. (DOC) [file pone.0044229.s010.doc]

**Supplementary Table 1**: Genome features of *B. asteroides* PRL2011

| **Trait** |  | **Number/value** |
| --- | --- | --- |
| Size (bp) |  | 2,167,301 |
| G+C content |  | 60.49% |
| Number of identified ORF |  | 1658 |
| Assigned function |  | 1326 |
| Unknown function |  | 332 |
| COG categories: |  | 1309 |
| - RNA processing and modification |  | 1 |
| - Energy production and conversion |  | 53 |
| - Cell cycle control, cell division, chromosome partitioning |  | 38 |
| - Amino acid transport and metabolism |  | 133 |
| - Nucleotide transport and metabolism |  | 55 |
| - Carbohydrate transport and metabolism |  | 186 |
| - Coenzyme transport and metabolism |  | 28 |
| - Lipid transport and metabolism |  | 34 |
| - Translation, ribosomal structure and biogenesis |  | 129 |
| - Transcription |  | 104 |
| - Replication, recombination and repair |  | 81 |
| - Cell wall/membrane/envelope biogenesis |  | 61 |
| - Posttranslational modification, protein turnover, chaperones |  | 47 |
| - Inorganic ion transport and metabolism |  | 57 |
| - Secondary metabolites biosynthesis, transport and catabolism |  | 5 |
| - General function prediction only |  | 110 |
| - Function unknown |  | 82 |
| - Signal transduction mechanisms |  | 40 |
| - Intracellular trafficking, secretion, and vesicular transport |  | 8 |
| - Defense mechanisms |  | 57 |
| Phage regions |  | 0 |
| IS transposase families: |  |  |
| IS256 |  | 5 |
| CRISPR |  | 1 |
| Fimbrial system type IV |  | 1 |
| Transporters: |  |  |
| - ABC systems |  | 44 |
| - PTS systems |  | 1 |
| - MFS |  | 32 |
