# Supplementary material for: Bifidobacterium asteroides PRL2011 Genome Analysis Reveals Clues for Colonization of the Insect Gut
Source: PLoS One. 2012 Sep 20;7(9):e44229. doi: 10.1371/journal.pone.0044229 (PMC3447821; doi:10.1371/journal.pone.0044229)
Supplement: Table S2 — ORFs identified on the B. asteroides PRL2011 genome which have no significant homology to other currently available bifidobacterial ORFs. (DOC) [file pone.0044229.s011.doc]

**Table S2**. ORFs identified on the *B. asteroides* PRL2011 genome which have no significant homology to other currently available bifidobacterial ORFs

| **ORFs** | **Predicted protein function** | **COG** |
| --- | --- | --- |
| BAST_0016 | alpha/beta hydrolase family protein | [R] |
| BAST_0026 | conserved hypothetical proteinwith SnoaL-like polyketide cyclase domain | [S] |
| BAST_0027 | transporter, probably Formate efflux permease | [P] |
| BAST_0036 | YheO-like protein | [S] |
| BAST_0040 | putative type III restriction enzyme |  |
| BAST_0042 | hypothetical protein |  |
| BAST_0043 | hypothetical protein |  |
| BAST_0044 | hypothetical protein |  |
| BAST_0045 | hypothetical protein |  |
| BAST_0059 | putative regulator of chromosome condensation, RCC1 | [DZ] |
| BAST_0077 | hypothetical protein |  |
| BAST_0078 | hypothetical protein |  |
| BAST_0087 | glycosyl hydrolase family 88 | [R] |
| BAST_0088 | putative BadF/BadG/BcrA/BcrD ATPase family protein |  |
| BAST_0093 | conserved hypothetical protein |  |
| BAST_0094 | AP endonuclease, family 2 | [G] |
| BAST_0096 | sugar-phosphate isomerase | [G] |
| BAST_0101 | conserved hypothetical protein | [E] |
| BAST_0102 | conserved hypothetical protein |  |
| BAST_0106 | hypothetical protein |  |
| BAST_0113 | hypothetical protein with YhhN-like protein domain |  |
| BAST_0114 | hypothetical protein |  |
| BAST_0123 | trehalose 6-phosphate synthase/phosphatase | [G] |
| BAST_0126 | hypothetical protein |  |
| BAST_0127 | hypothetical protein |  |
| BAST_0128 | hypothetical protein |  |
| BAST_0131 | putative permease, no TCDB homolog |  |
| BAST_0135 | hypothetical protein |  |
| BAST_0164 | putative lipase |  |
| BAST_0180 | hypothetical protein |  |
| BAST_0185 | hypothetical protein |  |
| BAST_0228 | hypothetical protein |  |
| BAST_0236 | hypothetical protein |  |
| BAST_0239 | alpha-glucuronidase | [G] |
| BAST_0244 | MFS transporter, probably The rhizopine related transporter, MocC | [GEPR] |
| BAST_0245 | hypothetical protein |  |
| BAST_0246 | conserved hypothetical protein with pyrimidine dimer DNA glycosylase |  |
| BAST_0251 | hypothetical protein |  |
| BAST_0255 | GNAT family acetyltransferase | [J] |
| BAST_0258 | putative transcriptional regulator | [K] |
| BAST_0260 | hypothetical protein with GDSL-like Lipase/Acylhydrolase domain |  |
| BAST_0262 | Catalase | [P] |
| BAST_0264 | DtxR family transcriptional regulator | [K] |
| BAST_0288 | NADH dehydrogenase, FAD-containing subunit | [C] |
| BAST_0289 | hypothetical protein |  |
| BAST_0290 | cydB, cytochrome d ubiquinol oxidase, subunit II | [C] |
| BAST_0293 | cydA, cytochrome d ubiquinol oxidase, subunit 1 | [C] |
| BAST_0315 | BadF/BadG/BcrA/BcrD ATPase family protein | [G] |
| BAST_0316 | P-loop ATPase |  |
| BAST_0318 | 2-dehydro-3-deoxyphosphogluconate aldolase/4-hydroxy-2-oxoglutarate aldolase | [G] |
| BAST_0322 | Fe-S oxidoreductase | [C] |
| BAST_0323 | iron-sulfur cluster binding protein | [C] |
| BAST_0324 | YkgG family protein | [S] |
| BAST_0353 | hypothetical protein |  |
| BAST_0354 | hypothetical protein |  |
| BAST_0356 | hypothetical protein |  |
| BAST_0357 | hypothetical protein |  |
| BAST_0358 | hypothetical protein |  |
| BAST_0369 | hypothetical protein |  |
| BAST_0370 | hypothetical protein |  |
| BAST_0380 | hypothetical protein | [R] |
| BAST_0382 | RCC1 repeat-containing protein | [DZ] |
| BAST_0422 | hypothetical protein with lipase (class 3) domain |  |
| BAST_0431 | hypothetical protein |  |
| BAST_0435 | RCC1 domain-containing protein | [DZ] |
| BAST_0436 | RCC1 domain-containing protein | [DZ] |
| BAST_0437 | hypothetical protein |  |
| BAST_0438 | RCC1 domain-containing protein | [DZ] |
| BAST_0439 | RCC1 domain-containing protein | [DZ] |
| BAST_0440 | RCC1 domain-containing protein | [DZ] |
| BAST_0443 | RCC1 domain-containing protein | [DZ] |
| BAST_0444 | RCC1 repeat-containing protein | [DZ] |
| BAST_0446 | RCC1 repeat-containing protein | [DZ] |
| BAST_0447 | RCC1 repeat-containing protein | [DZ] |
| BAST_0448 | hypothetical protein |  |
| BAST_0450 | ABC transporter, permease |  |
| BAST_0452 | RCC1 repeat-containing protein | [DZ] |
| BAST_0456 | hypothetical protein |  |
| BAST_0457 | hypothetical protein |  |
| BAST_0459 | hypothetical protein |  |
| BAST_0461 | transcriptional regulator, MarR family | [K] |
| BAST_0463 | methyltransferase type 12 | [QR] |
| BAST_0496 | aminobenzoyl-glutamate utilization protein B | [R] |
| BAST_0544 | transporter, probably Quaternary ammonium compound efflux pump | [P] |
| BAST_0550 | transporter, probably 2-oxoglutarate:malate antiporter | [P] |
| BAST_0556 | 6-phospho-beta-glucosidase | [G] |
| BAST_0582 | DedA family protein | [S] |
| BAST_0584 | ArsC family protein | [P] |
| BAST_0615 | RCC1 repeat-containing protein | [DZ] |
| BAST_0616 | RCC1 repeat-containing protein | [DZ] |
| BAST_0617 | hypothetical protein |  |
| BAST_0656 | hypothetical protein |  |
| BAST_0663 | transcription regulator | [K] |
| BAST_0669 | putative transcriptional regulator TetR family |  |
| BAST_0684 | hypothetical protein |  |
| BAST_0698 | transporter, probably The putative hydroxymethylpyrimidine transporter, CytX | [F] |
| BAST_0699 | ADP-ribosylglycohydrolase family protein | [O] |
| BAST_0750 | hypothetical protein |  |
| BAST_0752 | glycosyl transferase, family 39, Pseudo-gene | [M] |
| BAST_0753 | glycosyl transferase, family 39, Pseudo-gene |  |
| BAST_0754 | glycosyl transferase, family 39, Pseudo-gene | [M] |
| BAST_0755 | putative hydrolase | [I] |
| BAST_0762 | hypothetical protein |  |
| BAST_0770 | hypothetical protein |  |
| BAST_0772 | conserved hypothetical protein | [S] |
| BAST_0780 | ribosomal-protein-alanine acetyltransferase |  |
| BAST_0801 | YheO domain protein | [S] |
| BAST_0802 | RCC1 repeat-containing protein | [DZ] |
| BAST_0809 | ferrochelatase | [H] |
| BAST_0811 | putative heme peroxidase | [S] |
| BAST_0868 | hypothetical protein |  |
| BAST_0876 | hypothetical protein |  |
| BAST_0907 | hypothetical protein |  |
| BAST_0913 | hypothetical protein |  |
| BAST_0914 | hypothetical protein |  |
| BAST_0915 | hypothetical protein |  |
| BAST_0917 | conserved hypothetical protein |  |
| BAST_0927 | MFS transporter, The Unknown Major Facilitator-6 (UMF6) Family | [GEPR] |
| BAST_0928 | ABC transporter |  |
| BAST_0980 | hypothetical protein |  |
| BAST_0986 | similar to regulator of chromosome condensation RCC1 | [DZ] |
| BAST_0987 | hypothetical protein |  |
| BAST_0988 | similar to regulator of chromosome condensation RCC1 | [DZ] |
| BAST_0989 | hypothetical protein |  |
| BAST_0990 | similar to regulator of chromosome condensation RCC1 | [DZ] |
| BAST_0992 | similar to regulator of chromosome condensation RCC1 | [DZ] |
| BAST_1012 | putative ABC-2 type transporter, no TCDB homolog |  |
| BAST_1015 | hypothetical protein |  |
| BAST_1016 | hypothetical protein |  |
| BAST_1017 | hypothetical protein |  |
| BAST_1018 | hypothetical protein |  |
| BAST_1019 | hypothetical protein |  |
| BAST_1022 | hypothetical protein |  |
| BAST_1026 | hypothetical protein |  |
| BAST_1027 | hypothetical protein |  |
| BAST_1030 | hypothetical protein |  |
| BAST_1031 | hypothetical protein |  |
| BAST_1035 | hypothetical protein |  |
| BAST_1036 | hypothetical protein |  |
| BAST_1039 | hypothetical protein |  |
| BAST_1052 | putative DJ-1/PfpI family protein | [R] |
| BAST_1057 | superoxide dismutase | [P] |
| BAST_1065 | hypothetical protein with MFS_1 domain |  |
| BAST_1076 | hypothetical protein with predicted permease domain |  |
| BAST_1078 | hypothetical protein |  |
| BAST_1080 | hypothetical protein |  |
| BAST_1081 | RNA polymerase sigma-70 factor | [K] |
| BAST_1101 | hypothetical protein |  |
| BAST_1135 | hypothetical protein |  |
| BAST_1145 | ABC transporter, permease protein |  |
| BAST_1147 | ABC transporter, permease protein, |  |
| BAST_1168 | hypothetical protein |  |
| BAST_1170 | nitrite reductase, Pseudo-gene |  |
| BAST_1171 | hypothetical protein |  |
| BAST_1173 | galacturan 1,4-alpha-galacturonidase | [M] |
| BAST_1175 | putative nitrite reductase | [Q] |
| BAST_1176 | hypothetical protein |  |
| BAST_1188 | OsmC-like protein | [O] |
| BAST_1193 | hypothetical protein |  |
| BAST_1215 | hypothetical protein |  |
| BAST_1225 | GNAT family acetyltransferase | [KR] |
| BAST_1236 | YheO domain-containing protein | [S] |
| BAST_1256 | hypothetical protein |  |
| BAST_1264 | hypothetical protein |  |
| BAST_1265 | hypothetical protein |  |
| BAST_1282 | hypothetical protein |  |
| BAST_1285 | hypothetical protein |  |
| BAST_1287 | hypothetical protein |  |
| BAST_1291 | hypothetical protein |  |
| BAST_1299 | hypothetical protein |  |
| BAST_1302 | L-serine dehydratase, beta subunit | [E] |
| BAST_1329 | tetratricopeptide TPR_2 repeat protein | [R] |
| BAST_1364 | altronate dehydratase | [G] |
| BAST_1365 | pectinesterase | [G] |
| BAST_1375 | mannonate dehydratase | [G] |
| BAST_1377 | 2-dehydro-3-deoxyphosphogluconate aldolase/4-hydroxy-2-oxoglutarate aldolase | [G] |
| BAST_1400 | putative rhamnosidase |  |
| BAST_1401 | hypothetical protein |  |
| BAST_1421 | hypothetical protein |  |
| BAST_1422 | hypothetical protein |  |
| BAST_1423 | hypothetical protein |  |
| BAST_1424 | hypothetical protein |  |
| BAST_1436 | hypothetical protein |  |
| BAST_1438 | hypothetical protein |  |
| BAST_1484 | hypothetical protein with NgoFVII restriction endonuclease domain | [L] |
| BAST_1485 | DNA methyltransferase |  |
| BAST_1488 | hypothetical protein |  |
| BAST_1493 | conserved hypothetical protein |  |
| BAST_1501 | putative regulator of chromosome condensation, RCC1 | [DZ] |
| BAST_1504 | putative TetR family transcriptional regulator | [K] |
| BAST_1523 | hypothetical protein |  |
| BAST_1524 | hypothetical protein |  |
| BAST_1557 | ABC transporter integral membrane protein |  |
| BAST_1569 | hypothetical protein |  |
| BAST_1583 | hypothetical protein |  |
| BAST_1587 | hypothetical protein |  |
| BAST_1590 | ABC transporter, extracellular substrate binding protein, probably Coelichelin uptake porter (Iron Chelate Uptake) | [P] |
| BAST_1591 | ABC transporter, permease protein,  probably Coelichelin uptake porter (Iron Chelate Uptake) | [P] |
| BAST_1592 | ABC transporter, permease protein,  probably Coelichelin uptake porter (Iron Chelate Uptake) | [P] |
| BAST_1594 | FAD-binding 9 siderophore-interacting domain protein | [P] |
| BAST_1605 | hypothetical protein |  |
| BAST_1606 | conserved hypothetical protein | [E] |
| BAST_1607 | conserved hypothetical protein |  |
| BAST_1610 | hypothetical protein |  |
| BAST_1612 | hypothetical protein |  |
| BAST_1613 | hypothetical protein with tetratricopeptide repeat domain |  |
| BAST_1614 | hypothetical protein |  |
| BAST_1658 | conserved hypothetical protein |  |
| BAST_1664 | hypothetical protein |  |
| BAST_1665 | hypothetical protein |  |
| BAST_1666 | hypothetical protein |  |
| BAST_1687 | type I restriction-modification system | [V] |
| BAST_1688 | type I restriction system, restriction subunit | [V] |
| BAST_1699 | hypothetical protein, pseudo-gene |  |
| BAST_1700 | hypothetical protein |  |
| BAST_1701 | hypothetical protein, pseudo-gene |  |

The genes part of the putative respiratory chain are highlighted in grey.
