# Supplementary material for: Bifidobacterium asteroides PRL2011 Genome Analysis Reveals Clues for Colonization of the Insect Gut
Source: PLoS One. 2012 Sep 20;7(9):e44229. doi: 10.1371/journal.pone.0044229 (PMC3447821; doi:10.1371/journal.pone.0044229)
Supplement: Table S3 — Predicted secretome of B. asteroides PRL2011. (DOC) [file pone.0044229.s012.doc]

**Table S3. Predicted secretome of *B. asteroides* PRL2011**

| **ORF** | **Protein Function** |
| --- | --- |
| BAST_0015 | beta-glucosidase |
| BAST_0054 | ABC transporter, extracellular substrate binding protein |
| BAST_0059 | putative regulator of chromosome condensation, RCC1 |
| BAST_0068 | conserved hypothetical protein |
| BAST_0078 | hypothetical protein |
| BAST_0090 | ABC transporter, extracellular substrate binding protein |
| BAST_0140 | conserved hypothetical protein with bacterial Ig-like domain (group 4) domain |
| BAST_0148 | ABC transporter, extracellular substrate binding protein |
| BAST_0191 | ABC transporter, extracellular substrate binding protein |
| BAST_0202 | beta-xylosidase |
| BAST_0205 | putative glycoside hydrolase |
| BAST_0207 | ABC transporter, extracellular substrate binding protein |
| BAST_0216 | ABC transporter, extracellular substrate binding protein |
| BAST_0230 | putative ABC transporter permease, no TCDB homolog |
| BAST_0237 | ABC transporter, extracellular substrate binding protein |
| BAST_0243 | conserved repeat domain protein with Cna protein B-type domain |
| BAST_0283 | ABC transporter, extracellular substrate binding protein |
| BAST_0317 | MFS transporter, probably Galactonate transporter |
| BAST_0354 | hypothetical protein |
| BAST_0369 | hypothetical protein |
| BAST_0370 | hypothetical protein |
| BAST_0382 | RCC1 repeat-containing protein |
| BAST_0406 | hypothetical protein |
| BAST_0435 | RCC1 domain-containing protein |
| BAST_0436 | RCC1 domain-containing protein |
| BAST_0438 | RCC1 domain-containing protein |
| BAST_0439 | RCC1 domain-containing protein |
| BAST_0440 | RCC1 domain-containing protein |
| BAST_0443 | RCC1 domain-containing protein |
| BAST_0444 | RCC1 repeat-containing protein |
| BAST_0447 | RCC1 repeat-containing protein |
| BAST_0452 | RCC1 repeat-containing protein |
| BAST_0493 | conserved hypothetical protein |
| BAST_0509 | lipoprotein signal peptidase |
| BAST_0526 | conserved hypothetical protein with LysM domain |
| BAST_0532 | peptidoglycan synthetase, penicillin-binding protein 3 |
| BAST_0536 | UDP-N-acetylmuramoylalanine--D-glutamate ligase |
| BAST_0560 | ABC transporter, extracellular substrate binding protein |
| BAST_0562 | ABC transporter, extracellular substrate binding protein |
| BAST_0603 | ABC transporter, extracellular substrate binding protein acid (ALA) and heme |
| BAST_0604 | ABC transporter, extracellular substrate binding protein acid (ALA) and heme |
| BAST_0611 | hypothetical protein |
| BAST_0615 | RCC1 repeat-containing protein |
| BAST_0617 | hypothetical protein |
| BAST_0652 | ABC transporter, extracellular substrate binding protein |
| BAST_0660 | ABC transporter, extracellular substrate binding/permease protein |
| BAST_0704 | ABC transporter, extracellular substrate binding protein |
| BAST_0714 | M23 peptidase domain protein |
| BAST_0716 | conserved hypothetical protein |
| BAST_0767 | hypothetical protein |
| BAST_0773 | hypothetical protein |
| BAST_0802 | RCC1 repeat-containing protein |
| BAST_0855 | transporter, probably Hemolysin C, HlyC |
| BAST_0864 | conserved hypothetical protein |
| BAST_0868 | hypothetical protein |
| BAST_0872 | conserved hypothetical protein |
| BAST_0873 | LPXTG-motif cell wall anchor domain protein |
| BAST_0950 | conserved hypothetical protein with phosphoribosyl transferase domain |
| BAST_0953 | conserved hypothetical protein |
| BAST_0986 | similar to regulator of chromosome condensation RCC1 |
| BAST_0987 | hypothetical protein |
| BAST_0988 | similar to regulator of chromosome condensation RCC1 |
| BAST_0989 | hypothetical protein |
| BAST_0990 | similar to regulator of chromosome condensation RCC1 |
| BAST_0992 | similar to regulator of chromosome condensation RCC1 |
| BAST_1015 | hypothetical protein |
|  |  |
| BAST_1100 | putative cell surface elastin binding protein EbpS |
| BAST_1149 | ABC transporter, extracellular substrate binding protein |
| BAST_1150 | ABC transporter, extracellular substrate binding protein |
| BAST_1160 | ABC transporter, extracellular substrate binding protein |
| BAST_1200 | ABC transporter, extracellular substrate binding protein |
| BAST_1207 | ABC transporter, extracellular substrate binding protein |
| BAST_1209 | CHAP domain containing protein |
| BAST_1223 | hypothetical protein |
| BAST_1237 | ABC transporter, extracellular substrate binding protein |
| BAST_1254 | ABC transporter, extracellular substrate binding protein |
| BAST_1271 | NlpC/P60 family protein |
| BAST_1290 | conserved hypothetical membrane protein |
| BAST_1296 | peptidase, S51 family |
| BAST_1310 | ABC transporter, extracellular substrate binding protein |
| BAST_1365 | pectinesterase |
| BAST_1369 | ABC transporter, extracellular substrate binding protein |
| BAST_1404 | ABC transporter, extracellular substrate binding protein |
| BAST_1466 | conserved hypothetical protein |
| BAST_1468 | secreted peptidyl-prolyl cis-trans isomerase protein |
| BAST_1491 | conserved hypothetical protein |
| BAST_1499 | ABC transporter, extracellular substrate binding protein |
| BAST_1501 | putative regulator of chromosome condensation, RCC1 |
| BAST_1523 | hypothetical protein |
| BAST_1524 | hypothetical protein |
| BAST_1577 | hypothetical protein |
| BAST_1578 | hypothetical protein |
| BAST_1590 | ABC transporter, extracellular substrate binding protein |
| BAST_1618 | conserved hypothetical protein with FMN-binding domain |
| BAST_1623 | putative pathogen-specific surface antigen |
| BAST_1624 | high-affinity Fe2+/Pb2+ permease |
| BAST_1638 | ABC transporter, permease protein |
| BAST_1640 | ABC transporter, extracellular substrate binding protein |
| BAST_1656 | glycosyltransferase |
| BAST_1675 | dimethyladenosine transferase |
| BAST_1681 | conserved hypothetical protein |
