# Supplementary material for: Bifidobacterium asteroides PRL2011 Genome Analysis Reveals Clues for Colonization of the Insect Gut
Source: PLoS One. 2012 Sep 20;7(9):e44229. doi: 10.1371/journal.pone.0044229 (PMC3447821; doi:10.1371/journal.pone.0044229)
Supplement: Text S1 — The mobilome of B. asteroides genome, the secretome of B. asteroides PRL2011, stability of B. asteroides cultures in presence of hydrogen peroxide, evaluation of growth survival of B. asteroides cultures over time. (DOC) [file pone.0044229.s013.doc]

**Text S1**

**The mobilome of *B. asteroides* genome.** Analysis of G+C content, amino acid usage, BLASTP best-match and codon preference of the *B. asteroides* PRL2011 genome revealed various chromosomal regions that were presumed to be acquired by HGT (Fig. S5), encompassing a total of 114,919 bp (5.3 % of the *B. asteroides* PRL2011 genome), which is more than any other bifidobacterial genome so far sequenced. Notably, among these it is worth mentioning a locus encoding an exopolysaccharide/capsule (BAST_1655-1663), a cluster of six genes (BAST_0159-164), whose protein products are predicted to be involved in lipid metabolism (triacyl-glycerol metabolism), and two regions encompassing a type I R/M system (BAST_1687-1688) and a type III R/M system (BAST_0039-0040). Furthermore, the genome of *B. asteroides* PRL2011 harbors five insertion sequences (IS), all belonging to the IS256 family, sharing similarities with the actinobacterium *Arthrobacter arilaitensis*, but rarely detected in bifidobacterial genomes [1]. The prevalence of IS elements of *B. asteroides* PRL2011 is much lower compared to other sequenced bifidobacteria [1]. Another putative mobile element in the *B. asteroides* PRL2011 genome is represented by a Clustered of Regularly Interspersed Short Palindromic Repeats (CRISPR) locus (BAST_1428-BAST_1435), homologs of which are known to act as an RNA interference defense system against the invasion of foreign genetic material, in particular phages [2].

**The secretome of *B. asteroides* PRL2011**. Surface proteins are pivotal for sustaining microbe-microbe and microbe-host interactions, as well as for the interplay of microorganisms with their environment. Analysis of the predicted proteome of PRL2011 for the presence of signal peptide sequences revealed the presence of 103 putative secreted proteins (Table S3). A large proportion of these putative extracellular proteins are predicted to represent transporters for sugars, amino acids and metals (e.g., iron and cobalt), whereas just two proteins appear to be carbohydrate-degrading enzymes, being similar to a β-xylosidase (BAST_0202) and a β-glucosidase (BAST_0015).

**Stability of *B. asteroides* cultures in presence of hydrogen peroxide.** *B. asteroides* PRL2011 was cultivated in the presence of two different concentrations of hydrogen peroxide, which did not affect growth of this bacterium compared to cultivation in the absence of H_2_O_2_ (Fig. S9). In contrast, similar amounts of hydrogen peroxide dramatically hampered growth of other intestinal bifidobacteria such as *B. bifidum* PRL2010 as well as a typical microaerophilic bifidobacterial strain such as *Bifidobacterium animalis* subsp. *lactis* DSM10140 [3] (Fig. S9). Furthermore, the tolerance of PRL2011 to hydrogen peroxide expressed as a survival percentage was evaluated upon exposure of cell cultures to 0.002 % and 0.004 % of H_2_O_2_ in the substrate. Notably, no major changes in the viability of PRL2011 was noted, suggesting a high level of resistance to hydrogen peroxide of this strain as compared to other bifidobacterial strains tested, including a strictly anaerobic strain like *B. bifidum* PRL2010 and microaerophilic microorganism such as *B. animalis* subsp. *lactis* DSM10140, all of which suffer a significant reduction in viability upon exposure to H_2_O_2_ (Fig. S9).

**Evaluation of growth survival of *B. asteroides* cultures over time.** Previous studies involving *Lactococcus lactis* demonstrated a remarkable increase in long term stability when its metabolism was shifted from fermentation to respiration [4], probably because it leads to a cessation of lactic acid production and therefore a reduction of acid stress. Thus, the level of survival of *B. asteroides* PRL2011 upon storage at 4°C for one week of a PRL2011 culture from aerobic condition vs. anaerobic condition was investigated. As shown in Fig. S9, a slightly higher rate of survival was noticed for the cultures of *B. asteroides* PRL2011 maintained under aerobic vs. anaerobic incubation, suggesting a respiratory phenotype and/or a higher tolerance to oxygen.

1. Bottacini F, Medini D, Pavesi A, Turroni F, Foroni E, et al. (2010) Comparative genomics of the genus Bifidobacterium. Microbiology.

2. Barrangou R, Fremaux C, Deveau H, Richards M, Boyaval P, et al. (2007) CRISPR provides acquired resistance against viruses in prokaryotes. Science 315: 1709-1712.

3. Meile L, Ludwig W, Rueger U, Gut C, Kaufmann P, et al. (1997) *Bifidobacterium lactis* sp. nov, a moderately oxygen tolerant species isolated from fermented milk. Systematic and Applied Microbiology 20: 57-64.

4. Lechardeur D, Cesselin B, Fernandez A, Lamberet G, Garrigues C, et al. (2011) Using heme as an energy boost for lactic acid bacteria. Curr Opin Biotechnol 22: 143-149.
